# Impairment of Macroautophagy in Dopamine Neurons Has Opposing Effects on Parkinsonian Pathology and Behavior

**DOI:** 10.1016/j.celrep.2019.09.029

**Published:** 2019-10-22

**Authors:** Benjamin H.M. Hunn, Siv Vingill, Sarah Threlfell, Javier Alegre-Abarrategui, Morgane Magdelyns, Thierry Deltheil, Nora Bengoa-Vergniory, Peter L. Oliver, Milena Cioroch, Natalie M. Doig, David M. Bannerman, Stephanie J. Cragg, Richard Wade-Martins

**Affiliations:** 1 Oxford Parkinson’s Disease Centre, Department of Physiology, Anatomy and Genetics, University of Oxford, South Parks Road, Oxford OX1 3QX, UK; 2 Université Catholique de Louvain, Louvain-la-neuve, Region Wallone 1348, Belgium; 3 Department of Physiology, Anatomy and Genetics, University of Oxford, South Parks Road, Oxford OX1 3QX, UK; 4 Medical Research Council Harwell Institute, Harwell Campus, Oxfordshire OX11 0RD, UK; 5 Medical Research Council Brain Network Dynamics Unit, Department of Pharmacology, University of Oxford, Mansfield Road, Oxford OX1 3TH, UK; 6 Department of Experimental Psychology, University of Oxford, Oxford OX1 3TA, UK

**Keywords:** Parkinson’s disease, autophagy, pathology, dopamine, neurotransmission, behavior, mouse models

## Abstract

Parkinson’s disease (PD) is characterized by the death of dopamine neurons in the substantia nigra pars compacta (SNc) and accumulation of α-synuclein. Impaired autophagy has been implicated and activation of autophagy proposed as a treatment strategy. We generate a human α-synuclein-expressing mouse model of PD with macroautophagic failure in dopamine neurons to understand the interaction between impaired macroautophagy and α-synuclein. We find that impaired macroautophagy generates p62-positive inclusions and progressive neuron loss in the SNc. Despite this parkinsonian pathology, motor phenotypes accompanying human α-synuclein overexpression actually improve with impaired macroautophagy. Real-time fast-scan cyclic voltammetry reveals that macroautophagy impairment in dopamine neurons increases evoked extracellular concentrations of dopamine, reduces dopamine uptake, and relieves paired-stimulus depression. Our findings show that impaired macroautophagy paradoxically enhances dopamine neurotransmission, improving movement while worsening pathology, suggesting that changes to dopamine synapse function compensate for and conceal the underlying PD pathogenesis, with implications for therapies that target autophagy.

## Highlights

• Impaired macroautophagy in DA neurons leads to p62+ inclusions and DA neuron death
• Macroautophagy impairment increases evoked extracellular DA and reduces DA uptake
• Impaired macroautophagy improves human α-synuclein overexpression motor phenotypes
• Paradox of enhanced DA neurotransmission but increased neuropathology in PD model

## Graphical Abstract

**Figure:**
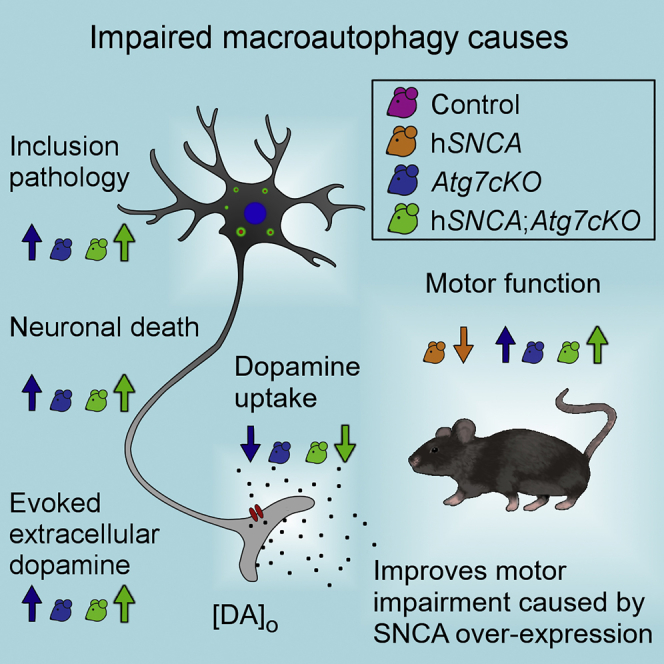

## Teaser

Hunn et al. use mouse models to uncover a complex relationship between macroautophagy, cellular neuropathology, dopamine neurotransmission, and Parkinsonian phenotypes. Paradoxically, impaired macroautophagy enhances dopamine neurotransmission and improves movement while worsening cellular pathology, with implications for therapies that target autophagy.

## Introduction

Parkinson’s disease (PD) is a neurodegenerative disorder characterized by the death of dopamine (DA) neurons in the substantia nigra pars compacta (SNc) and the accumulation of the pre-synaptic protein α-synuclein in Lewy bodies and Lewy neurites (Spillantini et al., 1997). PD typically manifests with bradykinesia, shortened gait, and resting tremor, along with non-motor symptoms including impaired cognition, constipation, and sleep disturbance (Chen et al., 2015). The human α-synuclein gene (*SNCA*) is mutated, duplicated, or triplicated in some familial forms of PD, and is the gene most significantly associated with sporadic PD in genome-wide association studies (Nalls et al., 2014, Singleton et al., 2003). Multiple studies have confirmed that accumulation of α-synuclein, including oligomeric species and fibrils, is deleterious to DA neurons and may help spread the disease throughout the nervous system (Bengoa-Vergniory et al., 2017, Brundin and Melki, 2017, Luk et al., 2012).

Macroautophagy is a process of cellular self-digestion by which cellular contents are degraded into their constituent parts (Harris and Rubinsztein, 2011). Perturbations in the autophagy-lysosome pathway (ALP) have been observed in the brains of sporadic PD patients, in induced pluripotent stem-cell-derived DA neurons from patients, and in rodent models (Chu et al., 2009, Fernandes et al., 2016, Hunn et al., 2015, Wallings et al., 2019). Many genes involved in both familial and sporadic PD encode proteins that participate in the ALP, including *LRRK2*, *GBA*, *VPS35*, *Parkin*, *PINK1*, and *ATP13A2* (Hunn et al., 2015). The ALP is considered to be one of the routes by which α-synuclein is cleared from the cell, and thus it has been proposed that perturbed macroautophagy may cause a toxic accumulation of α-synuclein and that stimulating macroautophagy may prevent or ameliorate this accumulation. Indeed, an autophagy enhancer (nilotinib) has already entered into a clinical trial for PD, and significant research effort is being expended on identifying potential drugs that may manipulate the ALP (Pagan et al., 2016).

In the present study, we generated transgenic mice with and without targeted macroautophagy impairment in DA neurons, both with and without overexpression of the human α-synuclein gene, to investigate how long-term inhibition of macroautophagy affects α-synuclein pathology and behavior in aged animals. We tested the hypothesis that macroautophagy impairment would worsen the pathological and behavior deficits associated with α-synuclein burden such that overexpression of α-synuclein, together with impairment of macroautophagy, would combine to produce a severe parkinsonian phenotype. Furthermore, we expected our data to confirm *in vitro* observations that impaired macroautophagy increases α-synuclein protein levels (Ebrahimi-Fakhari et al., 2011, Klucken et al., 2012, Lee et al., 2013, Webb et al., 2003). We found that impaired macroautophagy generated p62-positive inclusions resembling Lewy bodies in the midbrain and led to age-related neuron loss in the SNc. However, despite marked neuronal loss, motor phenotypes were unexpectedly improved as the impairment of macroautophagy led to increased evoked extracellular concentrations of DA and slowed DA uptake. Overall, our findings demonstrate that impaired macroautophagy enhances DA neurotransmission, improving movement and masking the cellular pathology, with implications for the treatment of PD.

## Results

### Impairment of DA Neuron Macroautophagy Exacerbates Parkinson’s Neuropathology in Transgenic Mice

**Figure 1:**
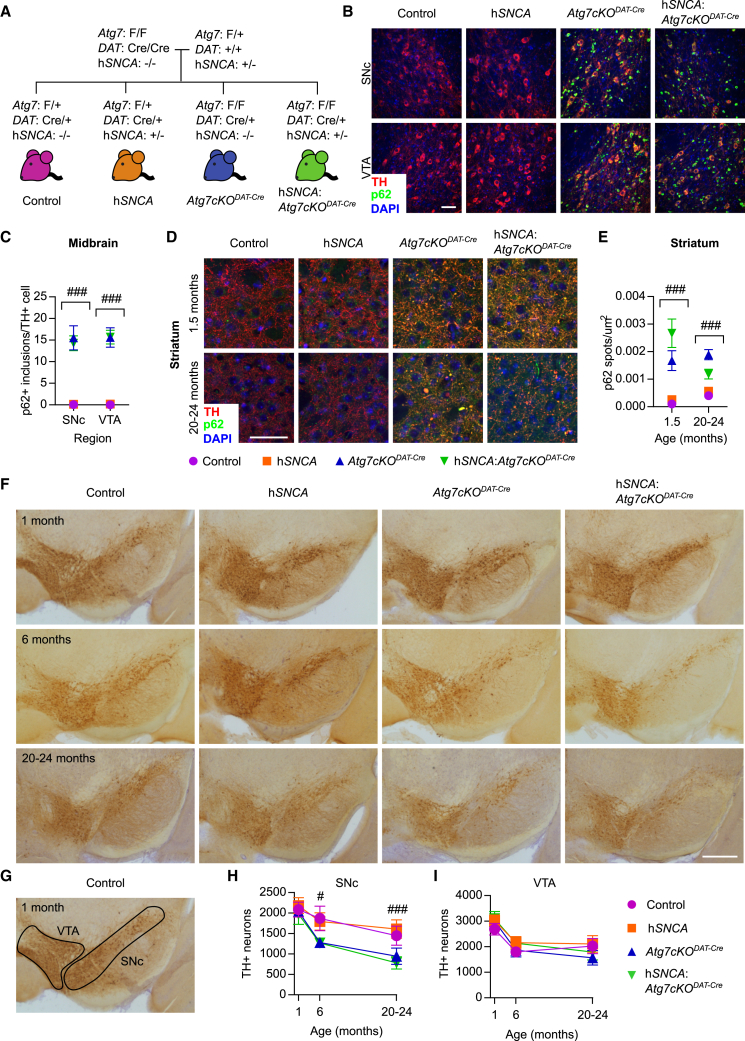
Macroautophagy Deficiency in DA Neurons Causes Selective Cell Loss in the SNc and the Formation of p62-Positive Lewy Body-Like Aggregates (A) Generation of experimental genotypes; all lines are heterozygous for *DAT-IRES-Cre*, with experimental cohorts either heterozygous (*Atg7 F/+)* or homozygous (*Atg7 F/F*) for floxed *Atg7* and the presence (h*SNCA+*) or absence (h*SNCA−*) of the human *SNCA* BAC transgene. All mice retained endogenous mouse α-synuclein. (B) p62 aggregates (green) were seen in SNc and VTA of 20–24-month-old *Atg7cKO*^*DAT-Cre*^ and h*SNCA:Atg7cKO*^*DAT-Cre*^ mice; TH (red) and 4′,6-diamidino-2-phenylindole (DAPI; blue). Scale bar, 50 μm. (C) Quantification of p62+ inclusions in the midbrain of 20–24-month-old mice. The *Atg7cKO*^*DAT-Cre*^ genotype showed an increased number of p62+ inclusions in both the SNc and VTA. Two-way ANOVA for *Atg7cKO*^*DAT-Cre*^ and h*SNCA.* Main effect of *Atg7cKO*^*DAT-Cre*^: increase in p62+ puncta in SNc and VTA, F_(1,8)_ = 82.15 and F_(1,8)_ = 125.3, respectively, ###p < 0.001, n = 3 animals per genotype; between 37 and 110 cells were analyzed per animal per region; mean ± SEM. (D) p62 aggregates (green) were seen in the dorsal striatum of 1.5- (upper panel) and 20–24-month-old (lower panel) mice carrying the *Atg7cKO*^*DAT-Cre*^ genotype; TH (red) and DAPI (blue). Scale bar, 50 μm. (E) Quantification of enlarged p62+ puncta in dorsal striatum of 1.5- and 20–24-month-old mice. The *Atg7cKO*^*DAT-Cre*^ genotype showed an increased number of p62+ enlarged puncta at both 1.5 and 20–24 months. Two-way ANOVA for *Atg7cKO*^*DAT-Cre*^ and h*SNCA*. Main effect of *Atg7cKO*^*DAT-Cre*^: increase in p62+ puncta at 1.5 and 20–24 months, F_(1,8)_:39.23 and F_(1,8)_:57.54, respectively, ###p < 0.001, n = 3 animals per genotype; 5–10 fields of view were analyzed per animal; mean ± SEM. (F) TH immunoreactivity on representative midbrain sections; scale bar, 500 μm. (G) Outlined region from (F) for quantitative analysis of TH+ neurons in VTA and SNc. (H and I) TH+ neurons in SNc (H) but not VTA (I) show an age-dependent loss associated with the *Atg7cKO*^*DAT-Cre*^ genotype (#p = 0.019, ###p < 0.001); n = 4–6 per genotype per age. Data are mean ± SEM. Detailed statistical analysis provided in STAR Methods. See also Figure S1.

We generated mice with a complete conditional deletion of the autophagy gene, *Atg7*, in DA neurons using a *DAT-IRES-Cre* system (*Atg7cKO*^*DAT-Cre*^), in the presence and absence of the human α-synuclein BAC transgene (h*SNCA*) (Figure 1A). Experimental genotypes were inherited in Mendelian ratios (Figure S1A), as expected, and weights did not differ by genotype (Figures S1B and S1C). Absence of the *Atg7* transcript in dopamine transporter (DAT)-positive neurons was confirmed using *in situ* hybridization (Figures S1D and S1E). Functional impairment of macroautophagy was demonstrated through accumulation of the macroautophagic substrate p62 in the SNc and ventral tegmental area (VTA) (Figure 1B). Almost all tyrosine hydroxylase (TH)-positive cells analyzed carried more than two p62-positive inclusions in both SNc and VTA in 20–24-month-old *Atg7cKO*^*DAT-Cre*^ (SNc, 96.6% ± 2.6%; VTA, 97.3% ± 1.6%; mean ± SEM; n = 3) and h*SNCA*:*Atg7cKO*^*DAT-Cre*^ (SNc, 96.5% ± 1.7%; VTA, 97.9% ± 0.4%; mean ± SEM; n = 3) animals. This was not seen in the midbrain of control (SNc, 0/243 TH+ cells; VTA, 0/277 TH+ cells; n = 3) or h*SNCA* (SNc, 0/214 TH+ cells; VTA, 1/256 TH+ cells; n = 3) animals. On average, the surviving DA neurons in aged mice contained approximately fifteen p62-positive inclusions in *Atg7cKO*^*DAT-Cre*^ animals (Figure 1C). Enlarged p62-positive puncta were also found in the dorsal striatum of both young (1.5 months) and old (20–24 months) animals (Figures 1D and 1E). In PD, TH-positive DA neurons of the SNc are preferentially lost, whereas the VTA is less affected (Hirsch et al., 1988, Rudow et al., 2008). In order to assess the region-specific effects of impaired macroautophagy and human α-synuclein expression on DA neuron number, blinded unbiased stereological cell counting of TH-positive neurons was performed on midbrain sections from all experimental genotypes at three different ages: 1, 6, and 20–24 months (Figures 1F–1I). We observed no loss of SNc neurons at 1 month of age, but a significant age-dependent loss at 6 and 20–24 months of age in *Atg7cKO*^*DAT-Cre*^ animals (Figure 1H). There was no association between h*SNCA* overexpression and SNc neuron loss in this model in the presence of the endogenous *Snca* gene, consistent with previous studies (Cabin et al., 2005, Masliah et al., 2000, Matsuoka et al., 2001, Tofaris et al., 2006) but in contrast to work in a *Snca*^*−/−*^ background (Janezic et al., 2013). In the VTA, neither *Atg7* deletion, nor h*SNCA* overexpression, was associated with neuron loss (Figure 1I).

**Figure 2:**
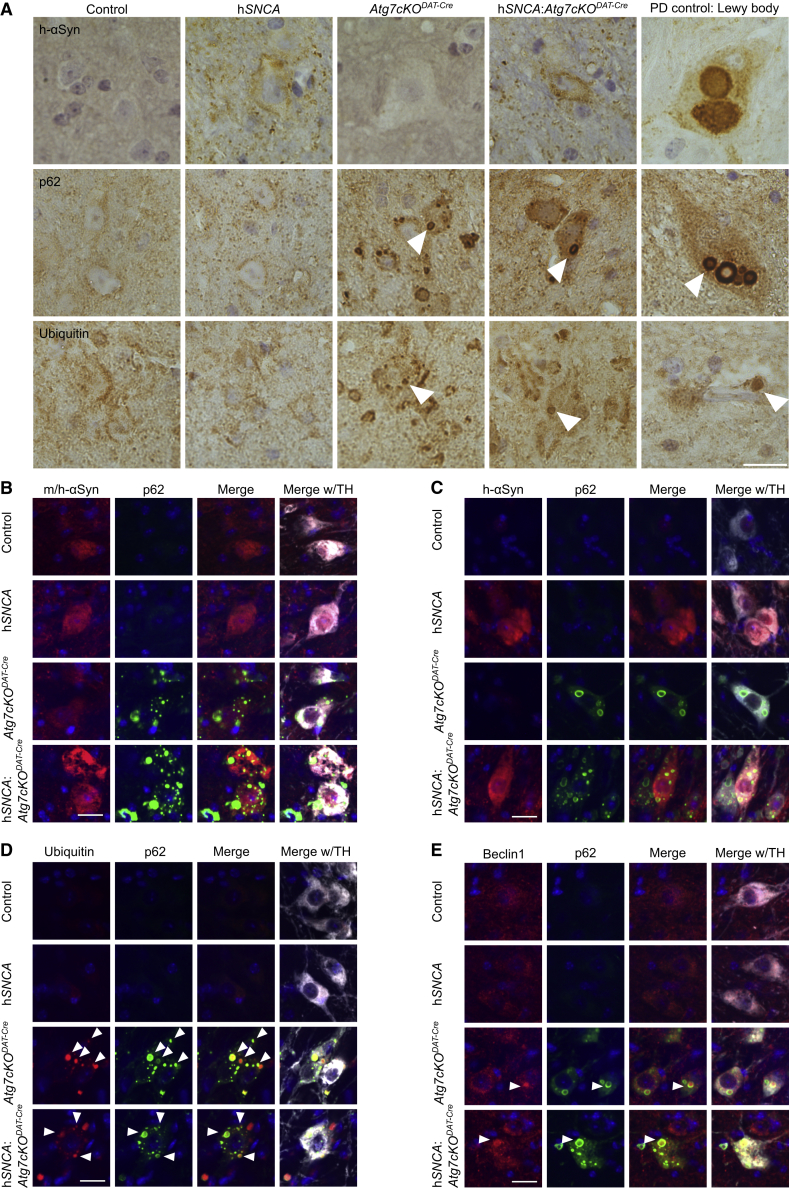
p62-Positive Lewy Body-Like Aggregates Co-localize with Ubiquitin and Beclin1, but Not with α-Synuclein (A) Immunohistochemistry in SNc of 20–24-month-old mice and human PD patients using antibodies against human α-synuclein, ubiquitin, and p62; scale bar, 20 μm. Arrowheads (middle panels), p62 inclusions; arrows (bottom panels), ubiquitin inclusions. (B–E) Representative images of fluorescence immunohistochemistry in 20–24-month-old mice of all genotypes. Sections stained with antibodies against (B) mouse/human α-synuclein, (C) human α-synuclein, (D) ubiquitin, and (E) beclin1 show co-localization of ubiquitin and the autophagic marker beclin1 with p62 aggregates. Scale bar, 50 μm. See also Figure S2.

The SNcs of mice aged 20–24 months were assessed for the presence of PD neuropathology (Figures 2A–2E). We observed clear overexpression of human α-synuclein in the h*SNCA* animals (Figures 2A–2C) and the presence of small donut-shaped p62 aggregates similar to the intracytoplasmic Lewy bodies found in human PD cases in the *Atg7cKO*^*DAT-Cre*^ mice (Figures 2A–2E). No α-synuclein-positive Lewy bodies or neurites were observed by staining with antibodies to either human/mouse (Figure 2B) or human-specific α-synuclein (Figures 2A and 2C). The p62-positive inclusions partially co-localized with markers for ubiquitin (Figures 2A and 2D) and beclin1 (Figure 2E), but showed little overlap with staining for the lysosomal marker Lamp1 (Figure S2A), the chaperone-mediated autophagy (CMA) marker Lamp2a (Figure S2B), or the autophagosome marker LC3b (Figure S2C), confirming that specifically targeting autophagy through the deletion of *Atg7* causes an accumulation of the phagophore and its ubiquitinated cargo early in the autophagy pathway. Almost all TH-positive cells analyzed carried more than two ubiquitin-positive inclusions in both SNc and VTA of 20–24-month-old *Atg7cKO*^*DAT-Cre*^ (SNc, 82.4% ± 4.5%; VTA, 90.7% ± 5.0%; mean ± SEM; n = 3) and h*SNCA*:*Atg7cKO*^*DAT-Cre*^ (SNc, 80.9% ± 6.2%; VTA, 87.0% ± 5.2%; mean ± SEM; n = 3) animals. This was not seen in in the midbrain of control animals (SNc, 8/243 TH+ cells; VTA, 0/277 TH+ cells; n = 3) or h*SNCA* (SNc, 4/214 TH+ cells; VTA, 4/256 TH+ cells; n = 3) animals. Similar inclusions positive for p62 (Figures S2D–S2I) but not α-synuclein (Figure S2D), and with clear overlap of the p62 staining with ubiquitin (Figure S2E) and Beclin1 (Figure S2F), but not with Lamp1 (Figure S2G), Lamp2a (Figure S2H), or LC3b (Figure S2I), were also seen in young animals.

**Figure 3:**
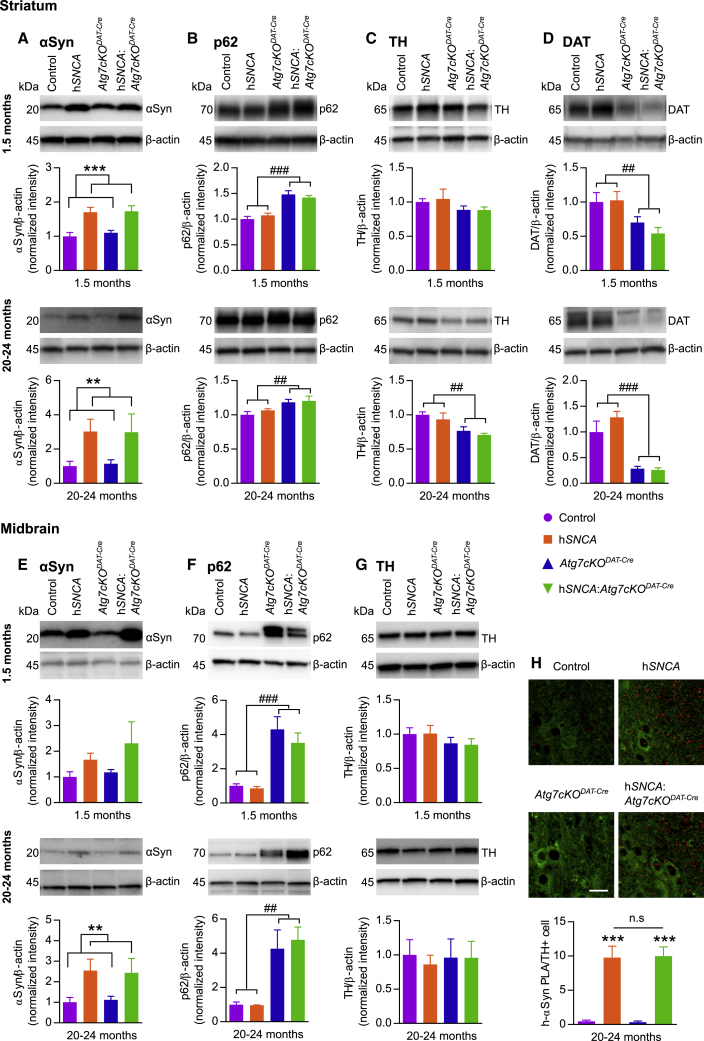
Decreased DAT and Increased p62 Expression in Striatum of *Atg7cKO*^*DAT-Cre*^ Animals (A–G) Immunoblots of DA neuronal proteins from CPu (A–D) and midbrain (E–G) of mice at 1.5 and 20–24 months of age were quantified and protein levels analyzed using two-way ANOVA for *Atg7cKO*^*DAT-Cre*^ and h*SNCA* at each time point. n = 3–5 animals per genotype. Protein amounts were first normalized to the β-actin loading control and then normalized to average of control animals for the respective age group. (A) In CPu h*SNCA* mice showed increased α-synuclein at 1.5 and 20–24 months of age (F_(1,18)_ = 27.37 and F_(1,12)_ = 13.30 respectively; ^∗∗∗^p < 0.001 and ^∗∗^p = 0.0033). (B) *Atg7cKO*^*DAT-Cre*^ mice showed increased p62 levels at 1.5 and 20–24 months of age (F_(1,18)_:52.89 and F_(1,12)_:9.957, respectively; ###p < 0.001, ##p = 0.0083). (C) At 20–24 months of age *Atg7cKO*^*DAT-Cre*^ mice showed a decrease in TH expression, consistent with neuronal loss (F_(1,12)_:14.30, ##p = 0.0026). (D) *Atg7cKO*^*DAT-Cre*^ mice showed decreased DAT levels at 1.5 and 20–24 months of age (F_(1,18)_:13.05 and F_(1,12)_:33.54, respectively; ##p = 0.002, ###p < 0.001). (E) In midbrain h*SNCA* mice showed increased α-synuclein at 20–24 months of age (F_(1,11)_ = 13.36, ^∗∗^p = 0.0038). (F) *Atg7cKO*^*DAT-Cre*^ mice showed increased p62 levels at 1.5 and 20–24 months (F_(1,18)_:32.19 and F_(1,11)_:17.89, respectively; ###p < 0.001, ##p = 0.0014). (G) TH: no significant changes were seen across genotypes at either age. (H) Representative images of PLA for human α-synuclein and quantification of PLA+ puncta per TH neuron. Scale bar, 20 μm. Two-way ANOVA for *Atg7cKO*^*DAT-Cre*^ and h*SNCA*. n = 3 animals per genotype; 25 TH+ve neurons were analyzed per animal. Presence of h*SNCA* showed significant increase of PLA signal over background (F_(1,8)_ = 77.8, ^∗∗∗^p < 0.001). Bonferroni *post hoc* test showed no difference between h*SNCA* and h*SNCA*:*Atg7cKO*^*DAT-Cre*^ genotypes. Data are mean ± SEM. See also Figures S3 and S4.

To define the levels of several key protein markers of PD pathology, quantitative immunoblotting was performed for levels of α-synuclein, p62, TH, and DAT in the striatum (Figures 3A–3D and S3A) and midbrain (Figures 3E–3G and S4A). α-Synuclein levels were elevated in mice expressing the h*SNCA* gene, as expected (Figures 3A, 3E, S3A, and S4A). However, there was no further increase of α-synuclein with impaired autophagy, suggesting that elevated α-synuclein is not cleared by macroautophagy in DA neurons *in vivo*. p62, an autophagic substrate, was increased in mice carrying the *Atg7cKO*^*DAT-Cre*^ genotype (Figures 3B and 3F) consistent with the accumulation of the phagophore following inhibition of autophagy. Furthermore, the *Atg7cKO*^*DAT-Cre*^ genotype was associated with a significant reduction of striatal TH in old animals (Figure 3C), and a significant reduction of DAT in the striatum of young and old animals (Figure 3D), corresponding to the PD-associated loss of DA neurons. We found no overall change in striatal (Figure S3) or midbrain (Figure S4) levels of autophagy markers beclin1 (Figures S3A and S4A) and LC3b (Figures S3B and S4B), lysosomal marker Lamp1 (Figures S3C and S4C), markers of vesicle-recycling Rab7 (Figures S3C and S4C), or CMA marker Lamp2a (Figures S3D and S4D). Although there are no α-synuclein aggregates present in these mice (Figures 2A–2C), we used our recently described α-synuclein proximity ligation assay (PLA) to detect significant levels of α-synuclein oligomers in the midbrain of mice expressing the h*SNCA* transgene aged to 20–24 months (Figure 3H). Notably, the number of oligomers does not change when autophagy is impaired, further arguing against macroautophagy as a major clearance mechanism for α-synuclein in this model.

### Impairment of Macroautophagy in DA Neurons Improves Parkinsonian Behavioral Phenotypes in Aged Transgenic Mice

**Figure 4:**
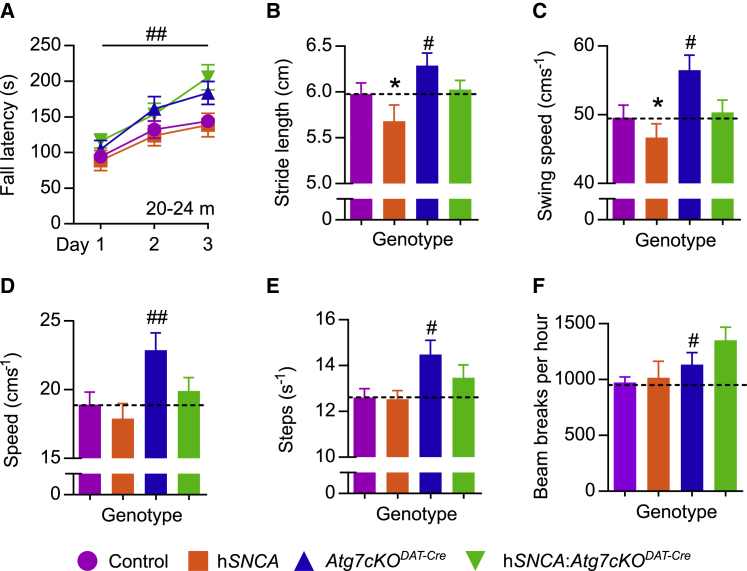
Macroautophagy Deficiency in DA Neurons Improves Parkinsonian Behavioral Phenotypes in 20–24-Month-Old Mice (A) Rotarod performance was enhanced in aged *Atg7cKO*^*DAT-Cre*^ mice when the mean of training days was considered (##p = 0.008). n = 9–17 per genotype. (B) Forefoot stride length was decreased in aged h*SNCA* mice (^∗^p = 0.041) but increased in aged *Atg7cKO*^*DAT-Cre*^ mice (#p = 0.018); n = 14–20 per genotype. (C) Forefoot swing speed was decreased in aged h*SNCA* mice (^∗^p = 0.027) but increased in aged *Atg7cKO*^*DAT-Cre*^ mice (#p = 0.011); n = 14–20 per genotype. (D) Gait velocity was increased in aged *Atg7cKO*^*DAT-Cre*^ mice (##p = 0.008); n = 14–20 per genotype. (E) Gait cadence was increased in aged *Atg7cKO*^*DAT-Cre*^ mice (#p = 0.014); n = 14–20 per genotype. (F) Locomotor activity was increased in *Atg7cKO*^*DAT-Cre*^ mice (#p = 0.019); n = 12–19 per genotype. All data are mean ± SEM. Detailed statistical analysis provided in STAR Methods. See also Figure S5.

We next determined the behavioral sequelae of these pathological changes. We subjected cohorts of young (3 months) and aged (20–24 months) transgenic mice to a battery of behavioral assays to test for the presence of motor and non-motor phenotypes that reflect symptoms of PD. *Atg7cKO*^*DAT-Cre*^ mice aged 3 months showed hyperlocomotor behavior (Figure S5F), in keeping with previous studies (Ahmed et al., 2012, Inoue et al., 2013), but no genotype-dependent differences in gait parameters or rotarod performance were seen (Figures S5A–S5E). Unexpectedly, we found that macroautophagic impairment in DA neurons improved multiple measures of Parkinsonian behaviors in aged mice. In aged mice, there were improvements in rotarod performance (Figure 4A), increased stride length (Figure 4B), attenuated bradykinesia (Figure 4C), increased velocity of spontaneous ambulation (Figure 4D), increased gait cadence (Figure 4E), and increased locomotor activity in the non-anxiogenic open field (Figure 4F). The expression of h*SNCA* was associated with reduced stride length (Figure 4B) and bradykinesia (Figure 4C), consistent with a mild Parkinsonian phenotype, which was improved in h*SNCA*:*Atg7cKO*^*DAT-Cre*^ mice.

### Evoked Extracellular DA Levels Are Increased When Macroautophagy Is Impaired in DA Neurons

**Figure 5:**
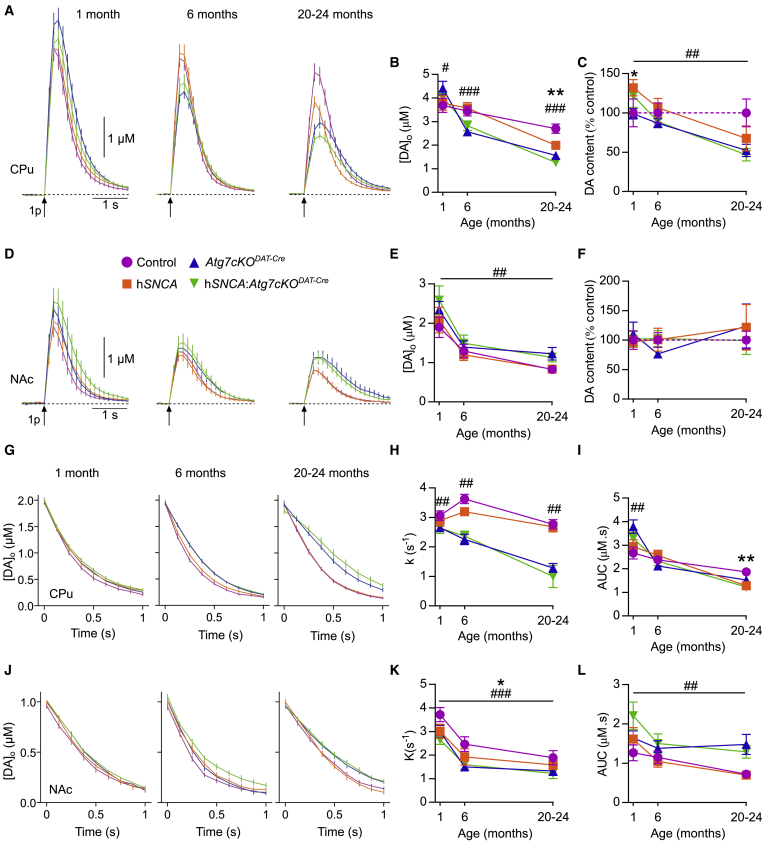
Macroautophagy Deficiency in DA Neurons Increases Extracellular DA Availability (A and D) Mean evoked [DA]_o_ profiles over time following single pulses in CPu (A) or NAc (D), in three age groups. All data were first analyzed with a three-way ANOVA for interaction between age, *Atg7cKO*, and h*SNCA* expression. Where interactions were present, the data were analyzed with a Bonferroni *post hoc* test at the respective time points. (B and E) Peak [DA]_o_ versus age in the CPu (B) and NAc (E). In CPu, 1 month, peak [DA]_o_ was increased in *Atg7cKO*^*DAT-Cre*^ (#p = 0.031). At 6 and 20–24 months, [DA]_o_ was reduced in *Atg7cKO*^*DAT-Cre*^ (###p < 0.001). [DA]_o_ was reduced in h*SNCA* at 20–24 months (^∗∗^p = 0.002); n = 32–40 from 4–5 mice per age per genotype. In NAc, *Atg7cKO*^*DAT-Cre*^ increased [DA]o independent of age (##p = 0.002); n = 16–20 from 4–5 mice per age per genotype. (C and F) DA content in the CPu (C) or NAc (F). In CPu at 1 month DA content was increased in h*SNCA* (^∗^p = 0.040). *Atg7cKO*^*DAT-Cre*^ mice had reduced DA content independent of age (##p = 0.013). In NAc, no genotype effects or interactions were observed. Values are percentage of age-matched control (dotted line); n = 4–5 per genotype per age. (G and J) [DA]_o_ versus time during decay after evoked release for DA transients matched at time = 0 s to 2 μM [DA] in CPu (G) and 1 μM in NAc (J). (H and K) Exponential decay constant (*k*) in the CPu (H) and NAc (K). *Atg7cKO*^*DAT-Cre*^ reduced *k* at all ages in both regions (##p < 0.01, ###p < 0.001). In NAc, h*SNCA* reduced *k*, independent of age (^∗^p = 0.043); CPu, n = 6-20, NAc, n = 9-12, from 4-5 mice per age per genotype. (I and L) Area under the [DA]_o_ curve (AUC) in CPu (I) and NAc (L). In CPu at 1 month, *Atg7cKO*^*DAT-Cre*^ increased AUC (##p = 0.002); at 20–24 months, h*SNCA* reduced AUC (^∗∗^p = 0.001); n = 32–40 from 4–5 mice per age per genotype. In NAc, *Atg7cKO*^*DAT-Cre*^ increased [DA]o AUC independent of age (###p < 0.001); n = 16–20 from 4–5 mice per age per genotype. Data are mean ± SEM. Detailed statistical analysis provided in STAR Methods. See also Figure S6.

We next sought to understand the factors underlying the divergent pathological and behavioral phenotypes we observed. We focused on understanding the outcome of macroautophagy impairment on DA neurotransmission, by monitoring extracellular DA ([DA]_o_) in real-time using fast-scan cyclic voltammetry (FCV) in striatal slices from mice aged 1, 6, and 20–24 months. In the caudate putamen (CPu), impaired macroautophagy in DA neurons was associated with increased peak levels of evoked [DA]_o_ following a single stimulation at 1 month, but decreased evoked [DA]_o_ at 6 or 20–24 months (Figures 5A and 5B). The reduced levels of evoked [DA]_o_ at 6 and 20–24 months are consistent with the SNc neuron loss in these age groups (see Figure 1H). The h*SNCA* transgene was associated with decreased evoked [DA]_o_ at 20–24 months in the CPu (Figures 5A and 5B). In the nucleus accumbens (NAc), there were increased peak levels of evoked [DA]_o_ associated with impaired macroautophagy at all ages (Figures 5D and 5E) despite no corresponding genotype-specific changes in VTA neuron number, which provide the primary DA input to NAc (see Figure 1I). The enhanced [DA]_o_ detected in CPu and NAc at 1 month prior to any loss of DA neurons in SNc is consistent with observations of a previous report (Hernandez et al., 2012), and suggests that additional processes are perturbed that lead to elevated evoked [DA]_o._ We assessed whether DA content of striatum was modified by impaired macroautophagy. DA content of CPu demonstrated an interaction in a three-way ANOVA between age and the h*SNCA* transgene, with pairwise comparisons revealing increased CPu DA in 1-month-old mice carrying the h*SNCA* transgene, but there was no effect of h*SNCA* in later age groups (Figure 5C). In contrast, the *Atg7cKO*^*DAT-Cre*^ genotype had reduced DA content of CPu, especially in old age (Figure 5C). The DA content of the NAc was unchanged (Figure 5F). These data broadly correspond with changes in DA neuron numbers (see Figures 1H and 1I) but do not explain the enhanced evoked [DA]_o_ detected with macroautophagy impairment either at young age in CPu or throughout life in NAc, suggesting other mechanisms regulating extracellular availability of DA are impacted by macroautophagy.

DA uptake via DAT is the major determinant governing [DA]_o_ (Giros et al., 1996) and we noted changes to the clearance kinetics of DA that suggested reductions to DA uptake. In particular, we noted that the falling phase of the [DA]_o_ curve appeared shallower in mice carrying the *Atg7cKO*^*DAT-Cre*^ genotype, particularly in old age (Figures 5A and 5D), consistent with the loss of DAT previously observed in this age group (Figure 3D). The prolonged clearance of DA associated with macroautophagy impairment was readily discernible when decay phases of curves were matched for DA concentrations (Figures 5G and 5J) (2 ± 0.2 μM in CPu and 1 ± 0.1 μM in NAc) to avoid confounding effects of DA concentration on Michaelis-Menten kinetics for which the uptake rate varies with substrate. We also calculated a DA uptake rate constant (*k*) using an approximation to exponential decay for each recording and identified significantly lower *k* values in mice carrying the *Atg7cKO*^*DAT-Cre*^ genotype in both CPu and NAc (Figures 5H and 5K). The DA uptake constant *k* was also reduced in NAc in mice positive for the h*SNCA* transgene (Figure 5K).

The biological activity of released DA at its receptors will be a function of its moment-by-moment extracellular concentration over time. *Atg7* cKO modifies both peak [DA]_o_ and its dynamic availability over time owing to changes in DA uptake. In order to determine the combined effects of reduced peak [DA]_o_ but diminished DA uptake on the net extracellular availability of DA (concentration × time), we calculated the area under the curve for [DA]_o_ evoked by a single stimulus pulse. In the CPu, the area under the [DA]_o_ curve was significantly increased in 1-month-old mice with the *Atg7cKO*^*DAT-Cre*^ genotype and was reduced in 20–24-month-old mice with the h*SNCA* genotype (Figure 5I). Notably, despite significant SNc neuron loss, the area under the curve was not different from control in mice carrying the *Atg7cKO*^*DAT-Cre*^ genotype, suggesting that the net effects of autophagic failure on lower levels of DA for release in CPu can be offset through reduced DA uptake. In NAc, the area under the [DA]_o_ curve was significantly increased when the *Atg7cKO*^*DAT-Cre*^ was present, across all ages (Figure 5L).

### Inhibition of Macroautophagy in DA Neurons Increases Evoked Extracellular DA Levels in Response to Subsequent Stimuli

**Figure 6:**
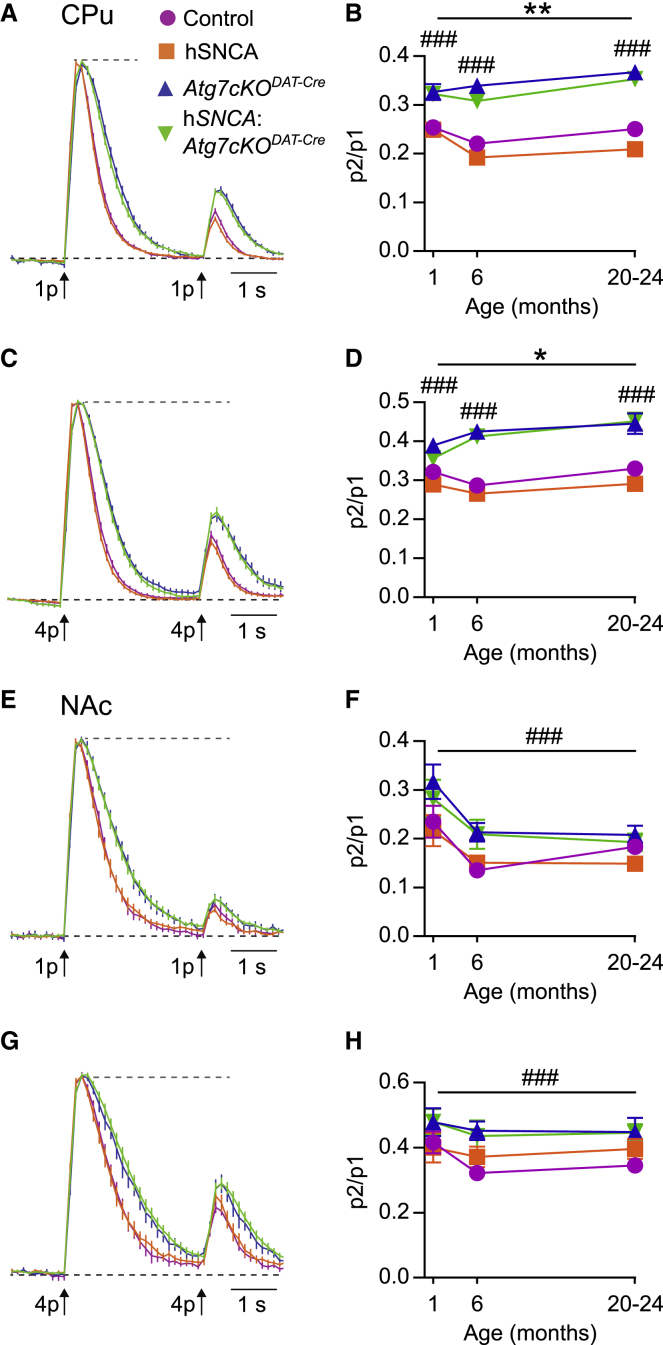
Macroautophagy Deficiency in Dopamine Neurons Increases [DA]o in Response to Subsequent Stimuli (A–H) Dopamine re-release was studied using paired stimuli. Left-sided panels (A, C, E, and G) demonstrate mean evoked [DA]_o_ profiles over time, and right-sided panels (B, D, F, and H) show [DA]_o_ following the second pulse as a proportion of the first pulse. Data were first analyzed with a three-way ANOVA for age, h*SNCA*, and *Atg7cKO*^*DAT*-*Cre*^. If an interaction was found between the age and genotype, each time point was analyzed separately. A line across all ages indicates differences independent of age. (A and B) Paired single pulses in the CPu of 20–24 month-old mice. Age-*Atg7cKO*^*DAT-Cre*^ interaction was found: *Atg7cKO*^*DAT-Cre*^ was associated with increased paired release at all ages (###p < 0.001). h*SNCA* was associated with diminished paired release, independent of age (^∗∗^p = 0.005). n = 32–40 from 4–5 mice per age group per genotype. (C and D) Paired trains of four pulses at 100 Hz in the CPu of 20–24-month-old mice. Age-*Atg7cKO*^*DAT-Cre*^ interaction was found: *Atg7cKO*^*DAT-Cre*^ was associated with increased paired release at all ages (###p < 0.001). h*SNCA* was associated with diminished paired release, independent of age (^∗^p = 0.039). (E and F) Paired single pulses in the NAc of 20–24-month-old mice. *Atg7cKO*^*DAT-Cre*^ increased paired release, independent of age (###p < 0.001). n = 16–20 from 4–5 mice per age group per genotype. (G and H) Paired trains of four pulses at 100 Hz in the CPu of 20–24-month-old mice. *Atg7cKO*^*DAT-Cre*^ increased paired release, independent of age (###p < 0.001). n = 16–20 from 4–5 mice per age group per genotype. Data are mean ± SEM. Detailed statistical analysis provided in STAR Methods.

Neurons of the SNc and VTA facilitate movement by acting in a coordinated manner of repeated firing in both tonic and phasic fashions. For this reason, it is important to also consider the ability of DA release sites to maintain release in response to subsequent stimuli. We used the paired-pulse method to assess the readiness of release sites for re-release, reflecting short-term dynamics of vesicle recruitment, as well as other mechanisms governing short-term synaptic plasticity (Abeliovich et al., 2000, Cragg, 2003). We used paired pulses and paired bursts (four pulses at 100 Hz) with a 3-s interval and explored paired-stimulation release ratios (P2/P1) across ages (Figures 6A–6H). DA release shows strong paired stimulus depression at this interval for both stimulus paradigms (Abeliovich et al., 2000, Cragg, 2003, Phillips et al., 2002, Schmitz et al., 2002). In the CPu, the presence of h*SNCA* reduced paired release (Figures 6B and 6D). Strikingly however, paired stimulus ratios were higher in mice with impaired macroautophagy in both CPu and NAc and across age groups (Figures 6B, 6D, 6F, and 6H), and for both stimuli, indicating that DA release at subsequent stimuli is improved when macroautophagy is impaired. This improved re-release, alongside diminished rates of DA uptake kinetics, together provide a neurochemical basis for the improved motor function seen in mice with impaired DA neuron macroautophagy.

We controlled for the potential changes to DAT that might arise through the use of the *DAT-IRES-Cre* expression system used here to delete *Atg7*, by also generating *Atg7* conditional deletion through the use of an alternative Cre driver line, the *TH-IRES-Cre* driver (Figures S6A and S6B). In *Atg7cKO*^*TH-Cre*^ mice, as in *Atg7cKO*^*DAT-Cre*^ mice, at 1 month of age we found enhanced evoked [DA]_o_ in CPu (Figures S6C and S6D) and NAc (Figures S6E and S6F); reduced *k* for DA uptake in CPu (Figures S6G and S6H), which was not significant in the NAc (Figures S6K and S6L); and enhanced paired release (Figures S6I, S6J, S6M, and S6N) without changes in underlying DA content (Figures S6Q and S6R), corroborating that the changes to DA transmission found are a result of impaired autophagy. Finally, analysis of [DA]_o_ to different stimulation frequencies found no genotype-specific effect (Figures S6O and S6P).

## Discussion

Here, we tested the hypothesis that combining overexpression of α-synuclein with impaired macroautophagy in DA neurons would lead to an enhanced model of age-related Parkinsonian pathology. We found no evidence for an increased burden of α-synuclein protein pathology with impaired macroautophagy in DA neurons. Instead, we unexpectedly found that impairment of macroautophagy in DA neurons has two divergent effects, causing a Parkinsonian neuropathology but paradoxically improving Parkinsonian behavior. The *Atg7cKO*^*DAT-Cre*^ genotype was associated with loss of SNc DA neurons and accumulation of p62 in surviving DA neurons. These are key neuropathological features found in PD and are consistent with previous similar studies (Ahmed et al., 2012, Friedman et al., 2012, Inoue et al., 2012). In general, extensive age-related nigral cell loss observed in *Atg7cKO*^*DAT-Cre*^ mice would be expected to result in Parkinsonian behavioral phenotypes, such as reduced locomotor activity, bradykinesia, and impaired rotarod performance. Instead, *Atg7cKO*^*DAT-Cre*^ mice exhibited improved motor function, concealing the Parkinsonian gait disturbance phenotypes shown in aged h*SNCA* transgenic mice. Our behavioral findings are consistent with a hyperdopaminergic state, suggesting perturbed DA neurotransmission underlies the behavioral findings.

The conditional knockout of *Atg7* driven using either *DAT-IRES-Cre* or *TH-IRES-Cre* lines was associated with an increase in peak evoked striatal [DA]_o_ where there was no loss of DA inputs, consistent with a previous study by Hernandez et al. (2012). Additionally, DA uptake was reduced, while paired stimulus release was enhanced, factors that will both enhance extracellular DA function. Taken together, these data suggest that impairment of macroautophagy has deleterious effects, particularly at the soma, driving cellular neuropathology and cell death, while at the same time having striatal effects that increase the extracellular availability of DA, mediating improvements in behavioral deficits. These results may explain why autophagic stimulation has been shown to ameliorate α-synuclein-related cellular pathology *in vivo*, but any improvements in behavioral phenotypes have been less clear (Sarkar et al., 2007, Spencer et al., 2009).

The deletion of *Atg7* in DA neurons led to a robust cellular inclusion pathology positive for p62 and ubiquitin partially co-staining with beclin1 across the midbrain, and to the death of neurons in the SNc. These observations of p62 inclusion pathology are consistent with the deletion of *Atg7* interrupting autophagy at the early stage at the formation of the phagophore, leading to accumulation of the phagophore and its ubiquitinated cargo. We see no change in LC3b levels or conversion of LC3b I to II, markers of later phases of autophagy associated with the maturation of the autophagosome. Previous studies have found either a decrease (Hernandez et al., 2012, Inoue et al., 2013) or increase in LC3 levels (Inoue et al., 2012) or lipidation and note that conversion of LC3b I to II across genotypes could be masked by non-dopaminergic cells in protein lysates. We found p62-positive axonal swellings in the striatum of our *Atg7cKO*^*DAT-Cre*^ animals by immunohistochemistry and clear accumulation of p62 in western blots of striatal tissue from both 1- and 24-month-old animals, suggesting that inclusion pathology is also present in dopaminergic terminals. In our analysis of cellular pathology, behavior, and DA neurotransmission, we studied cohorts of animals as “young” and as “old” as possible within the technical limitations of the respective methodologies, although a perfect age match between techniques was not always possible. Finally, our observation that impairment of macroautophagy does not increase accumulation of α-synuclein oligomers suggests that pathological α-synuclein is not substantially degraded by the autophagy pathway *in vivo*.

Our experiments show that impairment of macroautophagy targeted to DA neurons reduces DA uptake even in the absence of neuron loss, creating a hyperdopaminergic state most notably in the NAc, which likely increases locomotor behavior, masking the mild PD motor phenotype associated with the h*SNCA* transgene. Slowed DA uptake is likely to be mediated by the reduced levels of DAT protein observed in the striatum. The reduction in DAT protein levels in *Atg7cKO*^*DAT-Cre*^ mice could be attributable to early toxicity in the axonal arbor causing loss of DA terminals. TH levels were maintained at young age, suggesting TH may be upregulated to maintain DA production despite DA terminal loss. Alternatively, reductions in DAT protein levels may be mediated by some other mechanism, for example increased DAT protein degradation, reduced DAT protein expression, or sequestration of DAT in the neuronal soma. There is previous indirect evidence suggesting that impairment of macroautophagy reduces DAT availability. Niu et al. (2016) showed that *Atg7cKO*^*TH-Cre*^ mice were less susceptible to MPTP intoxication. Similarly, Hung et al. (2014) demonstrated that acute administration of *Atg7* small interfering RNA (siRNA) into the SNc reduced the toxicity of MPP+, the toxic metabolite of MPTP. Entry of MPP+ into DA neurons is mediated by DAT, and therefore a reduction in DAT availability would reduce MPTP toxicity. Both studies are consistent with our findings of reduced DAT activity in *Atg7cKO*^*DAT-Cre*^ mice.

The observation that *impaired* macroautophagy in DA neurons is associated with an *improvement* in motor performance masking motor phenotypes has implications for therapeutic trials in PD. Clinical trials are currently under way using nilotinib, a stimulator of macroautophagy, to treat PD (Pagan et al., 2016). Our work finds that macroautophagy does not play a major role in the clearance of α-synuclein protein in DA neurons *in vivo* and suggests that, although stimulating macroautophagy may be beneficial to cellular pathology and to long-term neuronal survival, it may also worsen motor performance via changes to striatal DA dynamics. This is a factor that should be considered in the design and interpretation of data from PD clinical trials, which typically rely heavily on scores of motor function to determine efficacy of experimental drugs.

## STAR★Methods

### Key Resources Table

**Table:** 

REAGENT or RESOURCE	SOURCE	IDENTIFIER
**Antibodies**		

Rabbit monoclonal anti-p62	Abcam	Cat#ab109012; RRID:AB_2810880
Mouse monoclonal anti-p62	Abcam	Cat#ab56416; RRID:AB_945626
Chicken polyclonal anti-TH	Abcam	Cat#ab76442; RRID:AB_1524535
Rabbit polyclonal anti-TH	Merck Millipore	Cat#AB152; RRID:AB_390204
Mouse monoclonal anti-human/mouse α-synuclein	BD Biosciences	Cat#610786; RRID:AB_398107
Rabbit monoclonal anti-human α-synuclein	Abcam	Cat#ab138501; RRID:AB_2537217
Mouse monoclonal anti-human α-synuclein	Abcam	Cat#ab80627; RRID: AB_1603277
Rabbit polyclonal anti-Beclin1	Abcam	Cat#ab62557; RRID:AB_955699
Rabbit polyclonal anti-Lamp1	Abcam	Cat#ab24170; RRID:AB_775978
Rabbit polyclonal anti-Lamp2a	Abcam	Cat#ab18528; RRID:AB_775981
Rabbit polyclonal anti-Ubiquitin	DAKO	Cat#Z0458; RRID:AB_2315524
Rabbit polyclonal anti-LC3b	Sigma	Cat#L7543; RRID:AB_796155
Rat monoclonal anti-DAT	Millipore	Cat#MAB369; RRID:AB_2190413
Rabbit monoclonal anti-Rab7	Abcam	Cat#ab137029; RRID:AB_2629474

**Biological Samples**		

Sections of substantia nigra from Parkinson’s disease patients	Parkinson’s UK Tissue Bank	https://www.parkinsons.org.uk/research/parkinsons-uk-brain-bank

**Experimental Models: Organisms/Strains**		

Mouse: B6.SJL-*Slc6a3*^*tm1.1(cre)Bkmn*^/J	The Jackson Laboratory	JAX: 006660
Mouse: B6.129X1-Th^tm1(cre)Te^/Kieg	European Mouse Mutant Archive	EM:00254
Mouse: B6.Cg-Atg7^tm1Tchi^	RIKEN BioResource Research Centre	RBRC02759
Mouse: B6.Cg-Tg(SNCA)OVX37Rwm *Snca*^*tm1Rosl*^/J	The Jackson Laboratory	JAX: 023837

**Software and Algorithms**		

SPSS Software, version 24	IBM	https://www.ibm.com/analytics/spss-statistics-software
GraphPad Prism 6	Graphpad Software	https://www.graphpad.com/
AxoScope 10.5	Molecular Devices	https://www.moleculardevices.com/
Strathclyde Whole Cell Program	University of Strathclyde	N/A

**Other**		

Noldus Catwalk	Noldus	https://www.noldus.com/animal-behavior-research/products/catwalk
Millar Voltameter	Julian Millar, Barts and the London School of Medicine and Dentistry	N/A

**Oligonucleotides**		

Primer sequences used for genotyping are listed in the STAR Methods section		

### Lead Contact and Materials Availability

Further information and requests for resources and reagents should be directed to the Lead Contact, Richard Wade-Martins (richard.wade-martins@dpag.ox.ac.uk). This study did not generate new unique reagents.

### Experimental Model and Subject Detail

All experiments and procedures conducted on animals were carried out in accordance with United Kingdom Home Office regulations under the *Animal (Scientific Procedures) Act* (1986), and were approved by the local ethical review board at the University of Oxford. All mice were housed in the University of Oxford Biomedical Services Building. Mice had access to standard food and water *ad libitum*. Mouse holding rooms were maintained at 22°C and 60 to 70% humidity on a 12 hour light-dark cycle. All mice were bred on a C57/BL6 background. For all experiments, animals of both sexes were used unless indicated otherwise. Mice of ages 1, 3, 6 and 20-24 months were used for experimental procedures as described in the Figure legends. Mice with a complete conditional deletion of the autophagy gene, *Atg7*, in DA neurons were generated using a *DAT-IRES-Cre* system (*Atg7cKO*^*DAT-Cre*^) or a *TH*-*IRES-Cre* system (*Atg7cKO*^*TH*-*Cre*^). *Atg7cKO*^*DAT-Cre*^ were subsequently cross-bred with a previously established human α-synuclein transgene (h*SNCA*) line (Janezic et al., 2013). All experiments and subsequent analyses were performed blind to genotype with the exception of immunoblots.

### Method Details

#### Genotyping of Experimental Animals

Mice were ear-clipped and the clip lysed in a 225 μL of lysis buffer (50 mM KCl, 1.5 mM MgCl, 10 mM Tris pH 8.5, 0.45% CA-630 (Igepal), 0.45% Tween-20, 225 ng Proteinase K) for 150 minutes at 55°C, followed by 20 minutes at 95°C to denature Proteinase K. Lysates were stored at 4°C. Genes of interest were amplified by PCR (see STAR Methods for primer sequences) and samples run on 2% agarose gels.

#### Transcardiac Perfusion

Mice were anaesthetised using 150 μL of pentobarbitone delivered via intraperitoneal injection. Following the cessation of toe pinch and palpebral reflexes, the thoracic cavity was opened and transcardiac perfusion performed with 25 mL of phosphate-buffered saline (PBS), followed by 25 mL of 4% paraformaldehyde (PFA) diluted in PBS. Brains were removed and fixed overnight in 4% PFA. For free-floating sections, brains were then cryoprotected in 30% sucrose for 3 days prior to sectioning. For paraffin sectioning, brains were kept in PFA for 14 days.

#### Preparation of Paraffin-Embedded Slices

PFA-fixed brains were paraffin-embedded using a Shandon Excelsior (Thermo Fisher Scientific, Massachusetts, United States of America). Brains were placed in the following solutions in a stepwise fashion, under vacuum conditions: 70% ethanol (90 minutes), 80% ethanol (90 minutes), 90% ethanol (90 minutes), 100% ethanol (3 steps of 90 minutes each), Histoclear (Thermo Fisher Scientific, Massachusetts, United States of America; 2 steps of 90 minutes each, and 1 step of 120 minutes), and finally paraffin wax (2 steps of 60 minutes and 1 step of 120 minutes). Paraffin blocks were sectioned at 4 μM using a Leica microtome (Leica Microsystems, Wetzlar, Germany), and sections mounted on slides.

#### Fluorescent Immunohistochemistry of Free-Floating Sections

Brains were immersed in dry ice and then sectioned in the coronal plane in 50 μm slices using a SM 2000R sliding microtome (Leica Microsystems, Wetzlar, Germany) with a cooled stage. Following sectioning, slices were stored in cryoprotectant at −20°C. On the day of staining, free floating sections were washed 3 times in PBS (GIBCO, 10010-015) with 0.02% TWEEN® 20, before blocking and permeabilization at RT. Sections were first incubated in PBS with 0.05% Tx100 (Sigma, T8787) for 30 min, then 10% normal donkey serum (Sigma, D9663) in PBS with 0.05% Tx100 for 1 h, followed by blocking with donkey α-mouse fAb fragment (Jackson ImmunoResearch, 715-007-003, 1:100) in PBS for 1h. Sections were then incubated with primary antibodies diluted in PBS with 0.01% TWEEN® 20 overnight at 4°C. Antibodies used for fluorescent IHC were p62 (abcam, ab109012, 1:2000), p62 (abcam, ab56416, 1:2000), TH (abcam, ab76442, 1:500), human/mouse α-synuclein (BD Biosciences, 610786, 1:250), human α-synuclein (abcam, ab138501, 1:250), Beclin1 (abcam, ab62557, 1:500), Lamp1 (abcam, ab24170, 1:500), Lamp2a (abcam, ab18528, 1:500), ubiquitin (DAKO, Z0458, 1:500) and LC3b (Sigma, L7543, 1:250). After three washes in 0.02% PBS-T, sections were incubated with secondary antibodies (Alexa Fluor conjugated donkey anti-mouse/rabbit; Invitrogen and Alexa Fluor conjugated donkey anti-chicken: Jackson Immunoresearch 1:500) and DAPI 1:5000 in PBS with 0.005% TWEEN® for 2h at RT. Sections were washed three times in PBS and mounted on glass slides (VWR, Superfrost® Plus) using FluorSave mounting medium (Millipore, Massachusetts, United States of America), coverslipped (VWR) and stored at 4°C. Slides were imaged using the Opera Phenix High Content Screening System (Perkin Elmer, Waltham, Massachusetts, United States). Brightness, contrast and image alignment were adjusted uniformly across genotypes. For co-localization staining with p62 and the other vesicular markers, 2-3 animals per genotype were stained and representative images chosen.

#### 3,3′-Diaminobenzidine Immunohistochemistry of Paraffin-Embedded Slices

Slides mounted with paraffin-embedded tissue were heated to 55°C to melt paraffin, and then rehydrated through sequential placement in xylenes, Histo-Clear (National Diagnostics, Georgia, United States of America), 100% ethanol, 95% ethanol, 70% ethanol and water. Slides were then immersed in 10% H_2_O_2_ for 30 minutes to quench endogenous peroxidases, and then rinsed in Milli-Q® water. In order to retrieve antigens masked during PFA fixation, slides were placed in citric acid buffer (Abcam, Cambridgeshire, United Kingdom) and microwaved for 10 minutes in total. Slides were washed 3 times with TBS and 0.05% TWEEN® 20, and then incubated with a blocking solution consisting of 10% goat serum in TBST for 1 hour. Slides were then incubated with a primary antibody (α-synuclein, 610786, BD Biosciences) p62 (ab109012, Abcam), Ubiquitin (Z0458, DAKO) in the blocking solution overnight. Slides were next washed 3 times with TBS and 0.05% TWEEN® 20, and then incubated for 1 hour with a species-appropriate biotin-conjugated secondary antibody (all manufactured by Vector Laboratories, Peterborough, United Kingdom) diluted 1:200 in the blocking solution. Slides were then washed 3 times with TBS and 0.05% TWEEN® 20. Subsequently, slides were incubated in an ABC solution (Vectastain® Elite® ABC Kit, manufactured by Vector Laboratories, Peterborough, United Kingdom) for 1 hour. Sections were then washed 3 times in TBS. Finally, sections were exposed to a solution consisting of 3,3′-diaminobenzidine (DAB) and urea hydrogen peroxide (SIGMAFAST kit, prepared as per the manufacturer’s instructions). Following DAB exposure, slides were again washed 3 times in TBS and 0.05% TWEEN® 20. Slides were then counterstained with hematoxylin (Gill’s formula, Vector Laboratories, Peterborough, United Kingdom), then rinsed in Milli-Q® water, and dehydrated with sequential submersion in 70% ethanol, 95% ethanol, 100% ethanol and 100% xylenes. Coverslips were mounted on slides using DPX mounting medium and imaged using an Evos FL Auto microscope (Life Technologies, California, United States of America). Brightness, contrast and image alignment were adjusted uniformly across genotypes.

#### Stereology

Brains were immersed in dry ice and then sectioned in the coronal plane in 50 μm slices using a SM 2000R sliding microtome (Leica Microsystems, Wetzlar, Germany) with a cooled stage. Following sectioning, slices were stored in cryoprotectant at −20°C. On the day of staining, free floating sections were washed 3 times in tris-buffered saline (TBS; 50 mM Tris, 150 mM NaCl, 2 mM KCl, pH 8), and then incubated in 3% H_2_O_2_ to quench endogenous peroxidases. Sections were then washed a further 3 times and then blocked for 1 hour in PBS with 10% goat serum. Sections were incubated in the blocking medium with an antibody against TH (ab152, Millipore) overnight at 4°C. The following day sections were washed 3 times in TBS, and then incubated with a biotinylated secondary antibody (Vector Laboratories, Peterborough, United Kingdom) diluted 1 in 200 in TBS with 0.1% Triton X-100 (TBST) for 1 hour. Following secondary antibody incubation, brain sections were washed 3 times in TBS. Subsequently, sections were incubated in an avidin-biotin complex (ABC) solution (Vectastain® Elite® ABC Kit, manufactured by Vector Laboratories, Peterborough, United Kingdom) for 1 hour. Sections were then washed 3 times in TBS. Finally, sections were exposed to a solution consisting of 3,3′-diaminobenzidine (DAB) and urea hydrogen peroxide (SIGMAFAST kit, prepared as per the manufacturer’s instructions). Following DAB exposure, sections were again washed 3 times in TBS. DAB-stained sections were transferred to microscopy slides (Superfrost Plus, VWR, Pennsylvania, United States of America), and slides were dried for 48 hours. Sections were then counterstained with hematoxylin (Gill’s formula, Vector Laboratories, Peterborough, United Kingdom), then rinsed in Milli-Q® water, and dehydrated with sequential submersion in 50% ethanol, 75% ethanol, 95% ethanol, 100% ethanol, 50% xylenes with 50% ethanol, and finally 100% xylenes. Coverslips were mounted on slides using DPX mounting medium. Unbiased stereological counting of TH-positive neurons was performed on every 3^rd^ section through the SNc, for a total of 8 sections per mouse. In each age group, mice of each genotype were processed together to prevent technical differences impacting neuron counts. TH positive neurons in the SNc and VTA were counted using an unbiased, online-counting optical fractionator method using Stereo Investigator software (MBF Bioscience, Vermont, United States of America) and a Zeiss Imager M2 microscope. The SNc and VTA were outlined using a 2x objective according to the mouse brain atlas provided by Paxinos and Franklin (2008). Neurons were subsequently counted at 100x. Pilot experiments with a 6 month-old control mouse were used to generate sampling parameters providing a Gundersen coefficient of error of less than 0.1. The parameters used were a sampling grid of 120 μm by 120 μm squares with a randomly placed counting frame of 60 μm by 60 μm, and an optical dissector height of 10 μm with a 2 μm guard distance.

#### Proximity Ligation Assay

Alpha-synuclein proximity ligation assay (AS-PLA) was performed as described previously (Roberts et al., 2019). Mice were overdosed with pentobarbitone 20% (w/v) and transcardially perfused with 4% (v/v) paraformaldehyde. Brains were dehydrated in graded alcohols and paraffin embedded, after which they were cut at 5 um. Sections were de-waxed in xylene and histoclear and then rehydrated in graded alcohols. Sections were then incubated in 3% H2O2 for 10 min followed by antigen retrieval in citrated buffer pH 6 (ab93678, Abcam). Sections were then blocked in 10% normal donkey serum, 1 M glycine, 0.1% Triton X-100 tris buffer saline for 1h RT, and were then incubated for 1h RT with anti-TH (ab152, Millipore). Sections were washed in tris buffer saline containing 0.05% Tween-20 (TBS-T) and then incubated for 1 h RT in AlexaFluor 488 (Life Technologies); from this point onward all steps were performed in the dark. Samples were then washed in TBS-T and incubated in Duolink PLA blocking buffer (Sigma) for 1 h 37 C, followed by an ON incubation at 4 C in probes in PLA diluent (Sigma, probes were generated with the syn-211 antibody, ab80627, Abcam). Samples were then washed with TBS-T and incubated with Duolink ligation reagents (Sigma) for 1h 37 C, washed with TBS-T and then incubated with Duolink amplification reagents (Sigma) for 2.5 h at 37 C. Samples were washed and mounted in FluorSave (Calbiochem). Fluorescent images were acquired with an EVOS FL auto system (Life Technologies) at 20x magnification. Intracellular PLA puncta were quantified manually after masking for TH positive cells. All image acquisition and counting were performed blind. For each animal, 4 random images were taken and analyzed in order to provide a representative sampling of the tissue. 25 TH+ve neurons were analyzed per animal. Counts are expressed as average PLA puncta per TH positive cell.

#### SDS-PAGE and Immunoblotting

For immunoblotting, mice were subjected to cervical dislocation, the brain removed and the following brain regions dissected: hindbrain, midbrain, hippocampus, cortex and striatum. Samples were then immediately frozen at −80°C. Protein samples were immersed in ice-cold RIPA buffer (20mM Tris, 150mM NaCl, 1% NP-40, 1% sodium deoxycholate, 0.1% SDS, 1 “complete mini protease inhibitor cocktail tablet,” deionised water) and subjected to mechanical disruption by a tissue homogenizer (Tissue-Tearor, Biospec Products) for ten seconds. Samples were centrifuged for 5 minutes at 12000 g, to leave only soluble protein. Protein quantification was performed using a bicinchoninic acid assay (Pierce BCA Protein Assay Kit) (Smith et al., 1985). 10 μg of protein samples were added to 6x sample buffer (375 mM Tris HCl pH 6.8, 30% mercaptoethanol, 12% SDS, 0.012% bromphenol blue, 60% glycerol) and loaded into a polyacrylamide gel, 4%–15% (Bio-Rad) before transfer to a PVDF membrane (Bio-Rad Trans-Blot Turbo). The membrane was blocked in 5% skimmed milk in TBS with 0.1% TWEEN® 20 for 1 hour. Following blocking, the membrane was incubated in primary antibody against human/mouse α-synuclein (610786, BD Biosciences, 1:500), DAT (MAB369, Millipore, 1:500), TH (ab152, Millipore, 1:2000) or p62 (ab109012, Abcam, 1:2000), p62 (abcam, ab56416, 1:2000), human α-synuclein (abcam, ab138501, 1:5000), Beclin1 (abcam, ab62557, 1:500), Lamp1 (abcam, ab24170, 1:2000), Lamp2a (abcam, ab18528, 1:2000), Rab7 (abcam, ab137029, 1:2000) and LC3b (Sigma, L7543, 1:5000) in blocking solution overnight at 4°C. The membrane was washed and probed with an HRP-conjugated secondary antibody (anti-mouse/anti-rabbit: Bio-Rad, anti-rat: Life Technologies) for one hour in blocking solution. Finally, the membrane was washed 3 times with TBS with 0.1% TWEEN® 20 and then developed using Immobilon Western Chemiluminescent HRP-substrate (Millipore) and imaged with a Chemidoc Touch (Bio-Rad).

#### Fast-Scan Cyclic Voltammetry

Fast-scan cyclic voltammetry (FCV) was performed as described by Threlfell et al. (2012). Experiments were performed in age- and sex-matched pairs of mice. Mice were killed by cervical dislocation and the brain removed into ice-cold artificial cerebrospinal fluid (aCSF) buffered by 4-(2-hydroxyethyl)-1-piperazineethanesulfonic (HEPES) acid and saturated with 95% O_2_ and 5% CO_2_. The composition of HEPES-buffered aCSF was 120 mM NaCl, 5 mM KCl, 20 mM NaHCO_3_, 6.7 mM HEPES acid, 3.3 mM HEPES salt, 2 mM CaCl_2_, 2 mM MgSO_4_, 1.2 mM KH_2_PO_4_ and 10 mM glucose. Coronal slices (300 μm thick) containing the striatum were prepared using a VT 1200S vibratome (Leica Microsystems, Wetzlar, Germany) in HEPES-buffered aCSF. Slices were then maintained in HEPES-buffered aCSF at room temperature with a constant O_2_ supply for one hour prior to experimentation. For DA recordings, striatal slices were placed into a chamber where a constant flow of aCSF was maintained at approximately 1.5 mL per minute at a temperature of 31 to 32°C. The composition of aCSF used for recording was 124 mM NaCl, 3.8 mM KCl, 26 mM NaHCO_3_, 2.4 mM CaCl_2_, 1.3 mM MgSO_4_, 1.2 mM KH_2_PO_4_ and 10 mM glucose, saturated with 95% O_2_ and 5% CO_2_. Prior to the start of recordings, slices were secured using silver pins and left to recover for a minimum of 20 minutes to allow both the brain slices to equilibrate at the recording bath temperature and the CFM to charge. A platinum/iridium concentric bipolar stimulating electrode of 25 μm diameter (FHC, Maine, USA) was placed at the surface of the striatal slice. A CFM was placed in the striatal slice at a depth of approximately 100 μm, approximately 150 μm away from the stimulating electrode. The CFM was connected to a Millar voltammeter (Julian Millar, Barts and the London School of Medicine and Dentistry, London, United Kingdom), and the voltammeter to an oscilloscope (Tektronix, Berkshire, United Kingdom). A scanning waveform was swept across the CFM by the voltammeter, and the resulting voltammogram was recorded on a computer connected to the oscilloscope, using either Strathclyde Whole Cell Program (University of Strathclyde, Glasgow, Scotland) or AxoScope 10.5 (Molecular Devices, California, United States of America). The scanning waveform passing across the CFM swept from −0.7 V to 1.3 V and to −0.7 V at a rate of 800 V/s, i.e., each scan took 5 ms. Voltammograms were recorded at a frequency of 8 Hz, and the CFM was switched out of circuit between scans. Electrical stimulation was delivered by the stimulating electrode for a duration of 200 μs at an amplitude of 600 μA. Electrical stimuli were delivered when the CFM was taken out of circuit, which precluded electrical stimuli from happening during a voltage sweep. Electrical stimuli were separated by 2.5 minutes to allow complete recovery of DA release. Following each experiment, electrodes were calibrated using 2 μM DA in aCSF. Six recording sites were used to examine DA neurotransmission: four sites were used in the caudate-putamen, and two sites in the nucleus accumbens. As in previous studies, data from the 2 nucleus accumbens sites were pooled, as were data from the 4 caudate-putamen sites, to provide accurate information regarding DA neurotransmission on average in each of these regions. In order to maximize the data obtained from each pair of mice, recordings were taken from two striatal slices per mouse per day at approximately ∼1.5 to 0.5 mm from Bregma in the anteroposterior plane. Cyclic voltammograms generated during calibration were used to determine the location of the DA oxidation potential to confirm the identity of the neurotransmitter being measured, sensitivity of the recording electrode. Calibration values were used with data acquired from acute striatal slices to determine changes in [DA]o over time. These data were applied to cyclic voltammograms generated from acute striatal slices to determine changes in [DA]_o_ over time. Data from cyclic voltammograms was exported to Microsoft Excel and processed using custom macros (S.J.C, unpublished data).

#### High-Performance Liquid Chromatography with Electrochemical Detection

Samples for high-performance liquid chromatograph (HPLC) with electrochemical detection were taken from the same striatal slices used for FCV experiments, immediately following the conclusion of FCV experiments, as performed previously (Taylor et al., 2014, Janezic et al., 2013, Sloan et al., 2016). From each striatal slice, 2 circular punches were taken from the caudate-putamen (diameter 2 mm, one from each hemisphere), and two circular punches were taken from the nucleus accumbens (diameter 1.2 mm, one form each hemisphere). Punches from the same slice and same region were stored together in 200 μL of 0.1M HClO_4_ at −80°C until an entire experimental set was complete and ready for analysis. Samples were then thawed at the same time for analysis and homogenized in HClO_4_ through sonication. Samples were then centrifuged at 15000 *g* for 15 minutes at 4°C to remove remnants of the cell membrane from the supernatant. The supernatant of caudate-putamen samples was diluted in 0.1M HClO_4_ at a ratio of 1:10 or 1:15 to ensure that all neurotransmitters were within the range of detection of the HPLC electrode. To optimize detection of neurotransmitters, HPLC mobile phase was used that contained 13% methanol, 0.8 mM ethylenediaminetetraacetic acid (EDTA), 0.5 mM octanesulfonic acid and 0.12 M NaH_2_PO_4_. 5 pmol (50 μL of 10^−7^ M) samples were injected into a C18 HPLC column (S150 × 4.6 mm; Agilent, California, USA) under high-pressure using an autosampler (AS-2057 PLUS, Jasco), at a flow rate of 1 mL per minute, over 15-20 minutes (adjusted to ensure overlap between different neurotransmitter peaks did not occur). Detection of neurotransmitters after they exited the column was performed by a Decade II SDS electrochemical detector (Antec, Zoeterwoude, the Netherlands) using a glassy carbon detecting electrode.

#### Behavioral Testing

All behavioral testing was performed in a blinded fashion. All tests were conducted in the light phase of the light-dark cycle. Two groups of mice were used: a young cohort with a mean age of 3 months (range 2.5 to 3.5 months), and an aged cohort with a mean age of 21 months (range 19 to 23 months).

##### Rota-rod

Mice were placed on a rota-rod apparatus (Med Associates, Vermont, United States of America) facing away from the experimenter. Mice were tested for 3 days on a rotating rod apparatus that accelerated from 4 to 40 rpm over 5 minutes. The latency to fall was recorded. If a mouse turned to face the experimenter or fell within the first 10 s of a trial then the trial was stopped, the mouse returned to the rod and the trial restarted from the beginning.

##### Locomotor Activity

Spontaneous locomotor activity was assessed by placing each mouse in a transparent plastic cage, and assessing locomotion by means of infra-red beam breaks over a 4-hour period in 30 minute intervals (apparatus manufactured by San Diego Instruments, California, United States of America). The floors of the cages were covered by a thin layer of saw dust, to a total volume of approximately 250 ml. Experiments were started between 0800 and 0900 hours.

##### Digital Gait Analysis

Mice were subjected to gait assessment using the CatWalk automated gait analysis system (Noldus Information Technology, Wageningen, the Netherlands). Mice were placed on a transparent glass platform that was cross-illuminated by a green light emitting diode. An orange contrasting light was placed over the mouse. Video recordings of 20 cm runs across the platform were made, and were accepted for analysis if they were between 0.5 and 5 s in duration and there was less than 35% variation in speed. The first 5 compliant runs were analyzed, or in the event of the mouse making 100 runs without reaching this target, all of the compliant runs within the first 100 were analyzed. Runs were analyzed using Catwalk software, and the average data for each mouse exported for analysis.

### Quantification and Statistical Analysis

Statistical analysis and presentation of data was performed using GraphPad Prism 6 for Macintosh (GraphPad Software, United States of America) and SPSS 24 (IBM, United States of America). For FCV, each observation was considered one n. Data were transformed as appropriate to reduce heteroscedasticity and analyzed using parametric tests, or non-parametric tests where these were suitable. Specific statistical tests and data transformations are reported in the text or as below:

Figure 1**. (H-I)** Three-way ANOVA for age, *Atg7cKO*^*DAT-Cre*^ and h*SNCA*. Data were log transformed for analysis. **(H)** In the SNc there was a significant age^∗^ *Atg7cKO*^*DAT-Cre*^ interaction (F(2,44) = 3.77, p = 0.031), and so the effect of *Atg7cKO*^*DAT-Cre*^ was examined at each level of age with a Bonferroni correction. At 1 month there was no effect of *Atg7cKO*^*DAT-Cre*^. At 6 and 20-24 months the *Atg7cKO*^*DAT-Cre*^ gene significantly decreased SNc DA neuron number (F(1,44) = 4.56, #p = 0.019 and F(1,44) = 19.99, ###p < 0.001, respectively). **(I)** There were no gene-specific effects in the VTA. n = 4-6 in each transgenic mouse line in each age group.

Figure 4**. (A)** Rotarod fall latency. Three-way mixed ANOVA for h*SNCA*, *Atg7cKO*^*DAT-Cre*^ and rotarod training day (as a within-subjects factor). There was a statistically significant interaction between training day and *Atg7cKO*^*DAT-Cre*^ (F(2,47) = 4.80, p = 0.010), with the *Atg7cKO*^*DAT-Cre*^ genotype performing significantly better on training day 3, but not on the other days (mean difference 53.04 s, 95% confidence intervals 23.51-82.57 s, p = 0.001, Bonferroni-adjusted). There was a significant between subjects effect of *Atg7cKO*^*DAT-Cre*^ improving rotarod performance when the average of all training days was considered (F(1,47) = 7.63, ##p = 0.008). n = 9-17 per genotype. Data are expressed as mean ± SEM **(B)** Forefoot stride length in aged mice. Two-way ANOVA for *Atg7cKO*^*DAT-Cre*^ and *hSNCA*. Main effect of *Atg7cKO*^*DAT-Cre*^ increasing stride length: F(1,64) = 5.91, #p = 0.018. Main effect of h*SNCA* reducing stride length: F(1,64) = 4.33, †p = 0.041. n = 14-20. Data are expressed as mean ± SEM **(C)** Forefoot swing speed in aged mice. Two-way ANOVA for *Atg7cKO*^*DAT-Cre*^ and h*SNCA*. Main effect of *Atg7cKO*^*DAT-Cre*^ increasing swing speed: F(1,64) = 6.92, #p = 0.011. Main effect of h*SNCA* decreasing swing speed: F(1,64) = 5.11, †p = 0.027. n = 14-20. Data were log-transformed for analysis but expressed here as raw mean ± SEM **(D)** Gait velocity in aged mice. Two-way ANOVA for *Atg7cKO*^*DAT-Cre*^ and h*SNCA*. Main effect of *Atg7cKO*^*DAT-Cre*^ increasing gait velocity: F(1,64) = 7.43, ##p = 0.008. n = 14-20. Data were log-transformed for analysis but expressed here as raw mean ± SEM **(E)** Gait cadence in aged mice. Two-way ANOVA for *Atg7cKO*^*DAT-Cre*^ and h*SNCA*. Main effect of *Atg7cKO*^*DAT-Cre*^ increasing gait cadence: F(1,64) = 6.33, #p = 0.014. n = 14-20. Data were log-transformed for analysis but expressed here as raw mean ± SEM **(F)** Locomotor activity in aged mice. Two-way ANOVA for *Atg7cKO*^*DAT-Cre*^ and h*SNCA*. Main effect of *Atg7cKO*^*DAT-Cre*^ increasing locomotion: F(1,53) = 5.89, #p = 0.019. n = 12-19. Data are expressed as mean ± SEM.

Figure 5**. (B)** Analysis of peak dopamine release in the caudate-putamen in three different age groups from traces shown in Figure 5A. Data were log transformed for analysis, but presented here as raw data. 3-way ANOVA for age, *Atg7cKO*^*DAT-Cre*^ and h*SNCA*. There was a two-way interaction between *Atg7cKO*^*DAT-Cre*^ and age: F(2,420) = 13.34, p < 0.001. Therefore, the effect of *Atg7cKO*^*DAT-Cre*^ was determined at each level of age, with a Bonferroni adjustment. At 1 month, raw peak [DA]o was 3.74 ± 0.16 μM in mice without *Atg7cKO*^*DAT-Cre*^ and 4.22 ± 0.16 μM in mice with *Atg7cKO*^*DAT-Cre*^, a difference of 0.48 (95% CI 0.044 to 0.91) μM (#p = 0.049). At 6 and 20-24 months, *Atg7cKO*^*DAT-Cre*^ was associated with reduced raw peak [DA]o of 0.81 (95% CI 0.40 to 1.22) and 0.93 (95% CI 0.54 to 1.32) μM, respectively, in both cases ###p < 0.001. There was a two-way interaction between h*SNCA* and age: F(2,420) = 4.36, p = 0.013. The effect of h*SNCA* was determined at each level of age with a Bonferonni correction. There was no effect *of* h*SNCA* at 1 or 6 months, but was significantly reduced at 20-24 months (††p = 0.002), with a raw difference of 0.50 μM (95% CI 0.11 to 0.89). n = 32-40 from 4-5 mice in each transgenic mouse line. Data are shown as mean ± SEM **(C)** Analysis of DA content in the CPu. 3-way ANOVA for age, *Atg7cKO*^*DAT-Cre*^ and h*SNCA*. There was an interaction between age and h*SNCA* F(2,42) = 3.603, p = 0.036. The effect of h*SNCA* was examined at each level of age, with a Bonferroni correction for multiple corrections. In the 1-month age group, the h*SNCA* genotype was associated with increased DA content (127.0% of control ± 9.2% in the h*SNCA* group and 99.5% ± 9.2% in the group without h*SNCA*). In other age groups there was no significant effect of h*SNCA*. Main effect of age, F(2,42) = 14.94, p < 0.001. Main effect of *Atg7cKO*^*DAT-Cre*^, F(1,42) = 6.73, p = 0.013. n = 4-5 per transgenic mouse line per age group. **(E)** Analysis of peak DA release in NAc in three different age groups from traces shown in Figure 5D. Data are square-root transformed but shown here as raw data for ease of comparison. 3-way ANOVA for age, *Atg7cKO*^*DAT-Cre*^ and h*SNCA*. Main effect of *Atg7cKO*^*DAT-Cre*^: F(1,204) = 9.84, ##p = 0.002. Main effect of age: F(2,204) = 42.46, p < 0.001. n = 16-20 from 4-5 mice in each age group in each transgenic mouse line. Data are shown as mean ± SEM **(F)** Analysis of dopamine content in the NAc. 3-way ANOVA for the effect of age, and *Atg7cKO*^*DAT-Cre*^ and h*SNCA*. n = 4-5 per transgenic mouse line per age group. Data expressed as mean ± SEM (M-O) Both *Atg7cKO*^*DAT-Cre*^ and h*SNCA* reduce DA reuptake rate in the NAc, independent of age. Mean [DA]o reuptake profiles in the NAc in three different age groups, concentration matched at 1 μM [DA]. Data from two sites in the NAc were pooled. Time 0 is when the falling phase of the dopamine transient passed closest to 1 μM [DA]. **(G)** *Atg7cKO*^*DAT-Cre*^ is associated with a reduced rate of caudate-putamen dopamine reuptake, independent of age. Mean [DA]o reuptake profiles in the caudate-putamen in three different age groups, concentration matched at 2 μM [DA]. Data from four sites in the CPu were pooled. Time 0 is when the falling phase of the dopamine transient passed closest to 2 μM [DA]. **(H)** Analysis of DA reuptake in the CPu in three different age groups from traces shown in Figure 5A. 3-way ANOVA for age, *Atg7cKO*^*DAT-Cre*^ and h*SNCA*. There was a 2-way interaction between *Atg7cKO*^*DAT-Cre*^ and age: F(2,322) = 9.74, p < 0.001. Therefore, the effect of *Atg7cKO*^*DAT-Cre*^ was determined at each level of age, with a Bonferroni adjustment. The *Atg7cKO*^*DAT-Cre*^ was significantly associated with slower dopamine reuptake at every level of age. ##p < 0.01. n = 6-20 from 4-5 mice in each age group in each transgenic mouse line. Data are shown as mean ± SEM **(I)** Analysis of area under the [DA]o curve (AUC) in the caudate-putamen in three different age groups from traces shown in Figure 5A. Data were log transformed for analysis, but presented here as raw data. 3-way ANOVA for age, *Atg7cKO*^*DAT-Cre*^ and h*SNCA*. There was a two-way interaction between *Atg7cKO*^*DAT-Cre*^ and age: F(2,420) = 7.25, p = 0.001. Therefore, the effect of *Atg7cKO*^*DAT-Cre*^ was determined at each level of age, with a Bonferroni adjustment. At 1 month, raw peak AUC was 0.39 ± 0.26 μMs^-1^ in mice without *Atg7cKO*^*DAT-Cre*^ and 0.50 ± 0.26 μMs^−^1 in mice with *Atg7cKO*^*DAT-Cre*^, a difference of 0.11 (95% CI 0.042 to 0.19) μMs^-1^ (##p = 0.002). At 6 and 20-24 months, *Atg7cKO*^*DAT-Cre*^ was not associated with any change in AUC. There was a two-way interaction between *hSNCA* and age: F(2,420) = 5.36, p = 0.005. The effect of *hSNCA* was determined at each level of age with a Bonferroni correction. There was no effect of h*SNCA* at 1 or 6 months, but was significantly reduced at 20-24 months (††p = 0.001), with a raw difference of 0.11 μMs^-1^ (95% CI 0.05 to 0.18). n = 32-40 from 4-5 mice in each transgenic mouse line. Data are shown as mean ± SEM **(K)** Analysis of DA reuptake in the NAc in three different age groups from traces shown in Figure 5D. Data were square root transformed for analysis, but presented here as raw data for ease of comparison. 3-way ANOVA for age, *Atg7cKO*^*DAT-Cre*^ and h*SNCA*. Main effect of age: F(2,139) = 43.10, p < 0.001. Main effect of *Atg7cKO*^*DAT-Cre*^: F(1,139) = 13.06, ###p < 0.001. Main effect of h*SNCA*: F(1,139) = 4.19, †p = 0.043 No other effects or interactions. n = 9-12 from 4-5 mice in each age group in each transgenic mouse line. **(L)** Analysis of [DA]o AUC in the NAc in three different age groups from traces shown in Figure 5D. Data are log transformed but shown here as raw data for ease of comparison. 3-way ANOVA for age, *Atg7cKO*^*DAT-Cre*^ and h*SNCA*. Main effect of *Atg7cKO*^*DAT-Cre*^: F(1,204) = 20.04, ###p < 0.001. Main effect of age: F(2,204) = 10.77, p < 0.001. n = 16-20 from 4-5 mice in each age group in each transgenic mouse line. Data are shown as mean ± SEM.

Figure 6**. (B)** Analysis of paired-pulse depression using single pulses in 3 different age groups. Three-way ANOVA for age, *Atg7cKO*^*DAT-Cre*^ and h*SNCA*. There was a two-way interaction between *Atg7cKO*^*DAT-Cre*^ and age: F(2,420) = 13.34, p < 0.001. At every age, *Atg7cKO*^*DAT-Cre*^ was associated with reduced paired-pulse depression (##p < 0.001, Bonferroni corrected). Main effect of age: F(2,419) = 6.54, p = 0.002. Main effect of h*SNCA*: F(1,419) = 7.88, p = 0.005. n = 32-40 from 4-5 mice in each age group in each transgenic mouse line. Data are shown as mean ± SEM **(D)** Analysis of paired-pulse depression using quadruple pulses at 100 Hz in 3 different age groups. Three-way ANOVA for age, *Atg7cKO*^*DAT-Cre*^ and h*SNCA*. There was a two-way interaction between *Atg7cKO*^*DAT-Cre*^ and age: F(2,418) = 5.06, p = 0.007. At every age, *Atg7cKO*^*DAT-Cre*^ was associated with reduced paired-pulse depression (##p < 0.001, Bonferroni corrected). Main effect of h*SNCA*: F(1,418) = 4.30, p = 0.039. n = 32-40 from 4-5 mice in each age group in each transgenic mouse line. Data are shown as mean ± SEM **(F)** Analysis of paired-pulse depression using single pulses in 3 different age groups. Data were log-transformed for analysis, but presented here as raw data for ease of comparison. Three-way ANOVA for age, *Atg7cKO*^*DAT-Cre*^ and h*SNCA*. Main effect of age: *F*_(2,204)_ = 9.00, p < 0.001. Main effect of *Atg7cKO*^*DAT-Cre*^: *F*_(1,204)_ = 17.38, ###p < 0.001. n = 16-20 from 4-5 mice in each age group in each transgenic mouse line. Data are shown as mean ± SEM (H) Analysis of paired-pulse depression in 3 different age groups. Three-way ANOVA for age, *Atg7cKO*^*DAT-Cre*^ and h*SNCA*. Main effect of *Atg7cKO*^*DAT-Cre*^: *F*_(1,204)_ = 16.32, ###p < 0.001. n = 16-20 from 4-5 mice in each age group in each transgenic mouse line. Data are shown as mean ± SEM.

### Data and Code Availability

The published article includes the datasets generated and analyzed during this study. No new code was generated during this study.

## Supplemental Information

Document S1. Figures S1–S6

Document S2. Article plus Supplemental Information
