# Supplementary material for: Impairment of Macroautophagy in Dopamine Neurons Has Opposing Effects on Parkinsonian Pathology and Behavior
Source: Cell Rep. 2019 Oct 22;29(4):920–931.e7. doi: 10.1016/j.celrep.2019.09.029 (PMC6856726; doi:10.1016/j.celrep.2019.09.029)
Supplement: Document S1. Figures S1–S6 [file mmc1.pdf]

Cell Reports, Volume *29*

Supplemental Information

Impairment of Macroautophagy in Dopamine Neurons Has Opposing Effects on Parkinsonian Pathology and Behavior

Benjamin H.M. Hunn, Siv Vingill, Sarah Threlfell, Javier Alegre-Abarrategui, Morgane Magdelyns, Thierry Deltheil, Nora Bengoa-Vergniory, Peter L. Oliver, Milena Cioroch, Natalie M. Doig, David M. Bannerman, Stephanie J. Cragg, and Richard Wade-Martins

Supplementary Figures

Hunn *et al.*, Figure S1

A

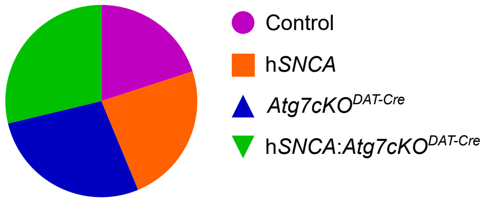

Genotype	Expected no.	Observed no.	Expected ratio	Observed ratio
Control	20	16	1	0.80
h*SNCA*	20	19	1	0.95
Atg7cKO$^{DAT\text{-}Cre}$	20	22	1	1.10
h*SNCA*:*Atg7cKO*$^{DAT\text{-}Cre}$	20	23	1	1.15
Total	80	80	4	4

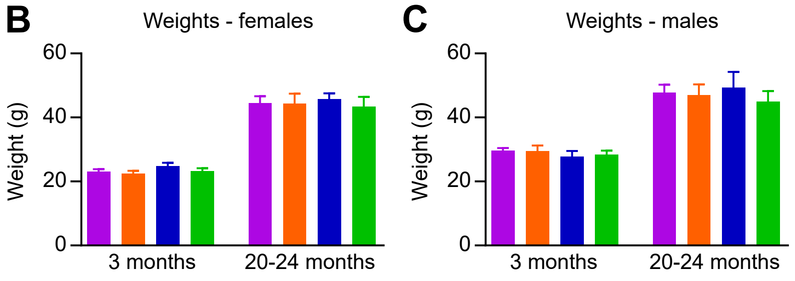

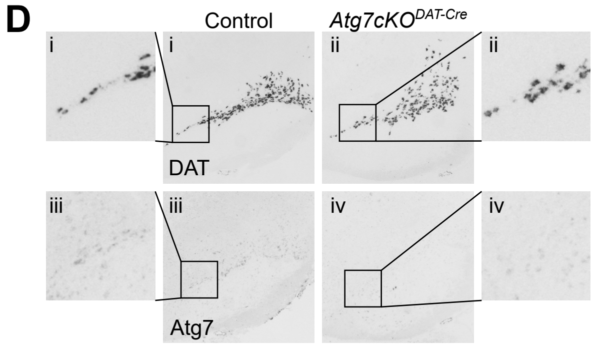

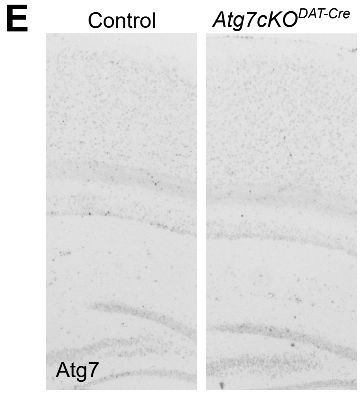

Figure S1. Genotype inheritance, weights and *in situ* hybridisation for *Atg7*. Related to Figure 1. **(A)** Mendelian inheritance ratios for each genotype for all litters from 3 experimental breeding pairs. No significant difference between expected and observed inheritance ratios (chi-square test, $\chi^2(2)=1.50$, $p=0.68$). (**B,C**) Mouse weight at two age points, for females (**B**) and males (**C**). Three-way ANOVA for age, *Atg7cKO*$^{DAT\text{-}Cre}$ and h*SNCA* genotypes: main effect of age, $F_{(1,110)}=278.784$, $p<0.001$. No other effects or interactions. Data are mean ± SEM. (**D**) Examples of *in situ* hybridisation to *DAT* (**i, ii**), or *Atg7* mRNA (**iii-iv**) in *Atg7cKO*$^{DAT\text{-}Cre}$ mice, in substantia nigra-ventral tegmental area (i-iv) where *Atg7* expression seen in control (***circled, iii***) is not detectable in cKO (***circled, iv***), and in hippocampus-cortex (**E**) where *Atg7* expression is preserved. In situ hybridisation (ISH) was performed on adjacent 15 μm sections.

Hunn *et al.*, Figure S2

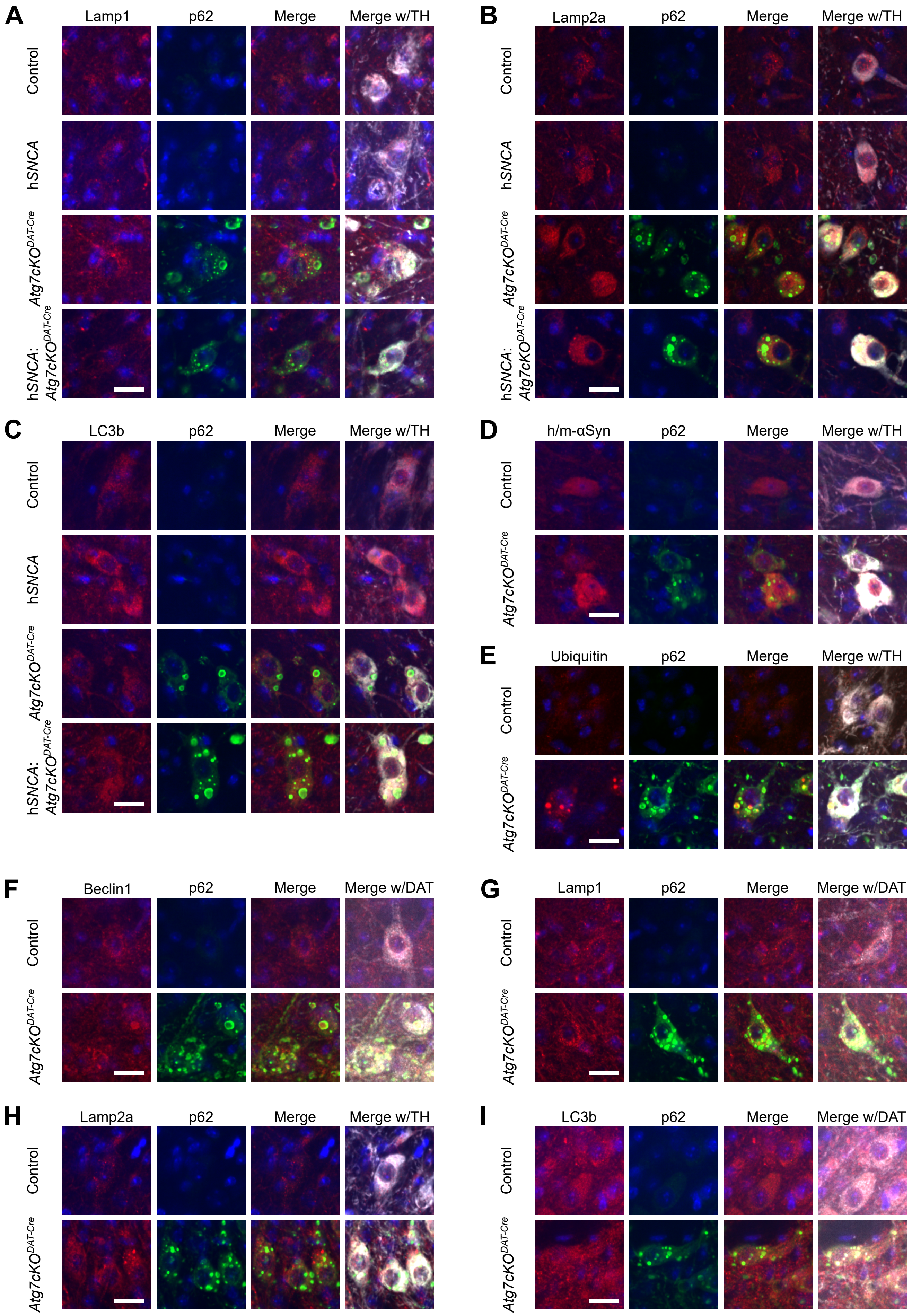

Figure S2. p62-positive Lewy body-like inclusions and markers of other cellular organelles. Related to Figure 2.

(**A-C**) Representative images of fluorescent immunohistochemistry in experimental cohort of 20-24 months old mice of all experimental genotypes, sections stained with antibodies against (**A**) Lamp1, (**B**) Lamp2a and (**C**) LC3b. (**D-I**) Representative images of fluorescent immunohistochemistry in experimental cohort of 1.5 months old mice of Control and $Atg7cKO^{DAT\text{-}Cre}$ genotypes, sections stained with antibodies against (**D**) mouse/human α-synuclein, (**E**) ubiquitin, (**F**) Beclin1, (**G**) Lamp1, (**H**) Lamp2a and (**I**) LC3b. All images counter-stained with p62 (green), DAPI (blue) and TH/DAT (grey). Scale bar 50 μm.

Hunn *et al.*, Figure S3

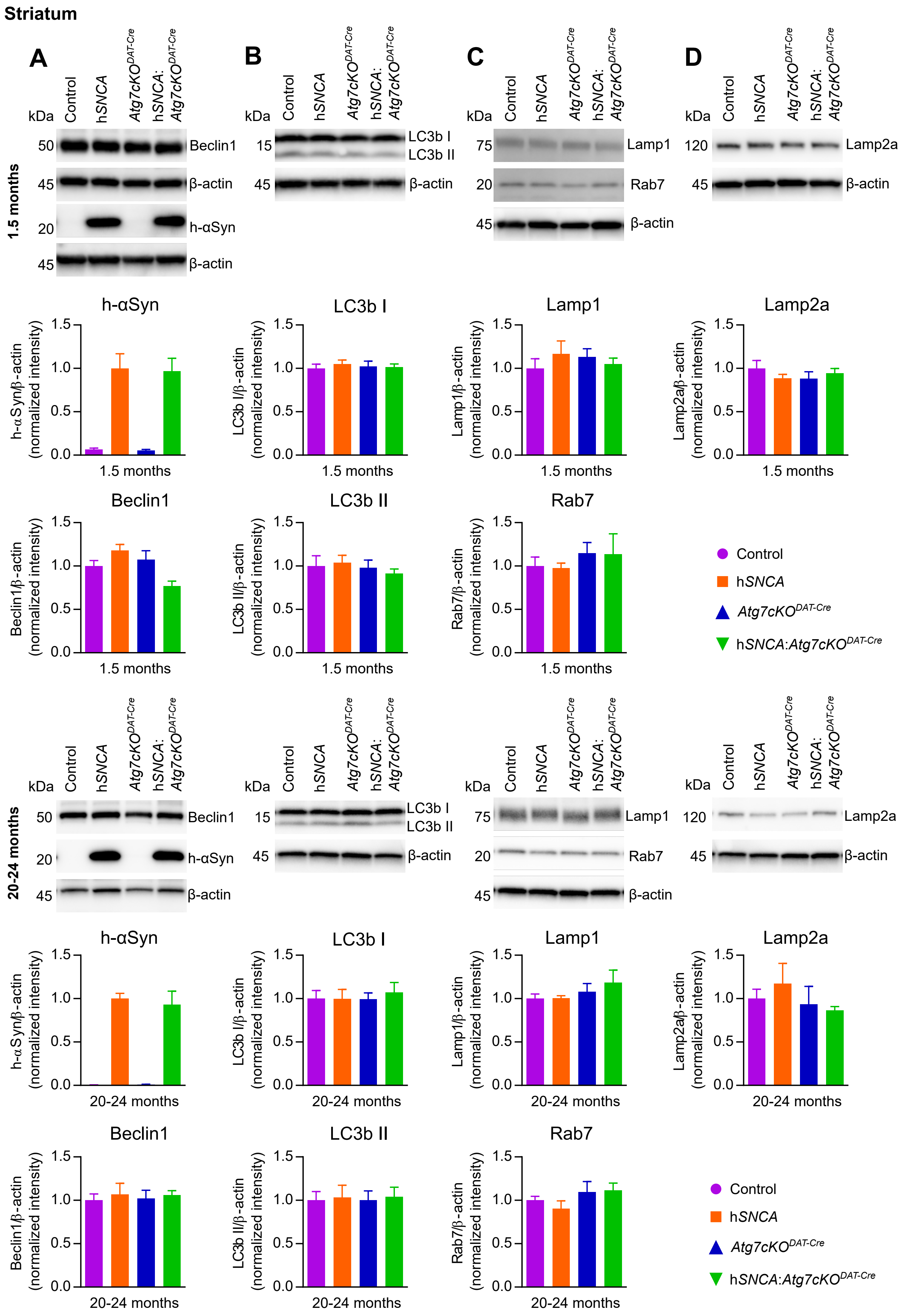

Figure S3. The levels of vesicular markers are unchanged in the striatum. Related to Figure 3. (**A-D**) Typical immunoblots of vesicular protein markers with quantification in CPu at 1.5 (upper panels) and 20-24 months (lower panels) old animals. (**A**) Human α-synuclein levels were analysed using t-test for *Atg7*cKO$^{DAT\text{-}cre}$ between h*SNCA* animals due to the lack of protein in m*SNCA* animals. There were no significant change across genotypes at either age (upper graphs) (**A-D**) Protein levels were analysed using two-way ANOVA for *Atg7*cKO$^{DAT\text{-}cre}$ and h*SNCA*. There were no significant changes across genotypes at either age found for (**A**) Beclin1 (lower graph), (**B**) LC3b I and II, (**C**) Lamp1 or Rab7 or (**D**) Lamp2a. Protein amounts were first normalized to the β-actin loading control and then normalized to average of control animals for the respective age group. Data are mean ± SEM.

Hunn *et al.*, Figure S4

Midbrain

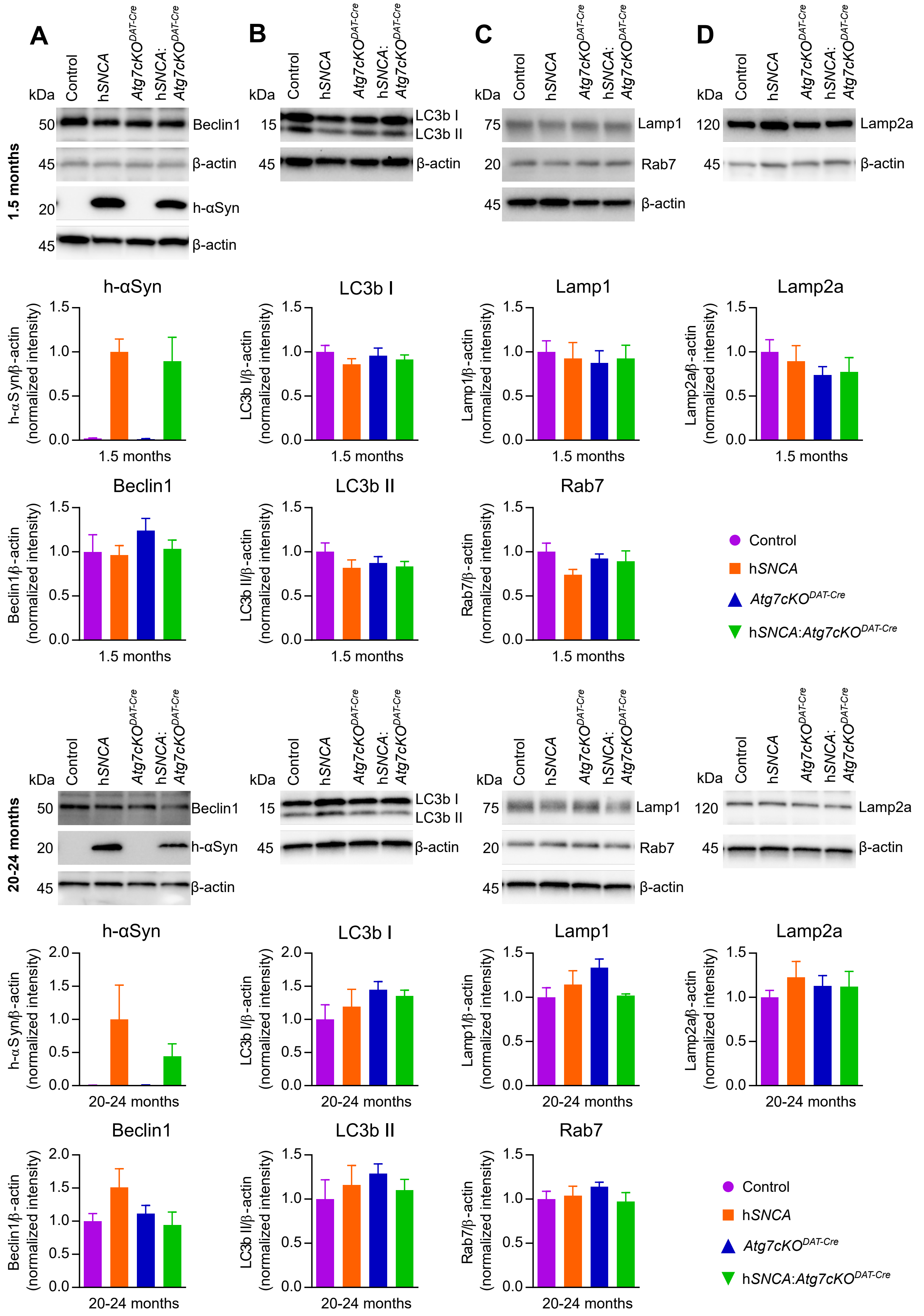

Figure S4. The levels of vesicular markers are unchanged in the midbrain. Related to Figure 3.

(**A-D**) Typical immunoblots of vesicular protein markers with quantification in midbrain at 1.5 (upper panels) and 20-24 months (lower panels) old animals. (**A**) Human α-synuclein levels were analysed using t-test for *Atg7*cKO$^{DAT\text{-}cre}$ between h*SNCA* animals due to the lack of protein in m*SNCA* animals. There were no significant change across genotypes at either age (upper graphs) (**A-D**) Protein levels were analysed using two-way ANOVA for *Atg7*cKO$^{DAT\text{-}cre}$ and h*SNCA*. There were no significant changes across genotypes at either age found for (**A**) Beclin1 (lower graph), (**B**) LC3b I and II, (**C**) Lamp1 or Rab7 or (**D**) Lamp2a. Protein amounts were first normalized to the β-actin loading control and then normalized to average of control animals for the respective age group. Data are mean ± SEM.

Hunn *et al.*, Figure S5

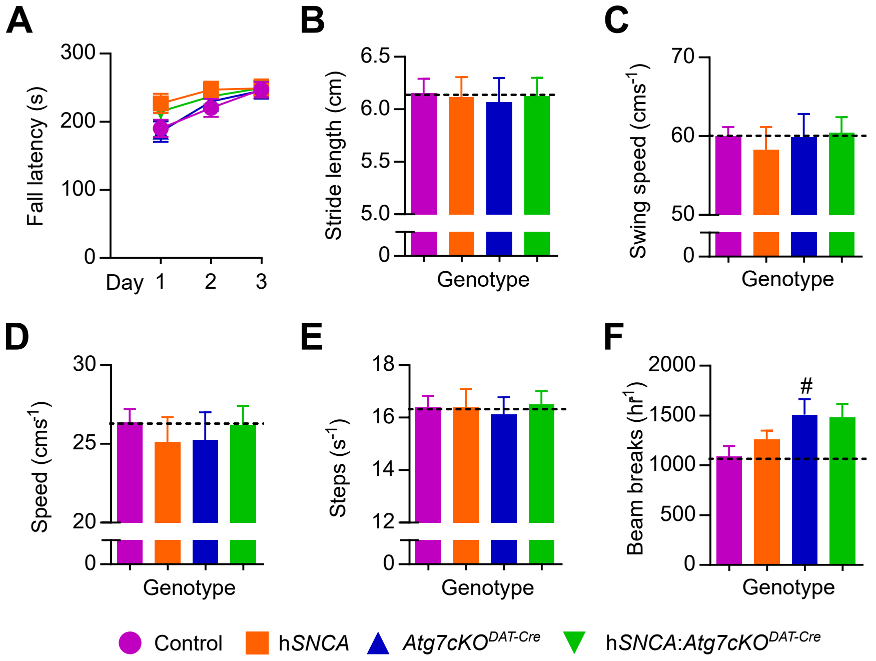

Figure S5. Hyperlocomotion, but no gait phenotypes or changes in rotarod performance, at 3 months in *Atg7cKO$^{DAT\text{-}Cre}$* mice. Related to Figure 4.

(A) Rotarod fall latency vs day of testing. Three-way mixed ANOVA for hSNCA, *Atg7cKO$^{DAT\text{-}Cre}$* and rotarod training day (as a within-subjects factor): no gene-specific effects or interactions. n=12-20 per genotype. **(B)** Forefoot stride length. Two-way ANOVA for *Atg7cKO$^{DAT\text{-}Cre}$* and h*SNCA*: no gene-specific effects or interactions. n=10-15 per genotype. **(C)** Forefoot swing speed. Two-way ANOVA for *Atg7cKO$^{DAT\text{-}Cre}$* and h*SNCA:* no gene-specific effects or interactions. n=10-15 per genotype. **(D)** Gait velocity. Two-way ANOVA for *Atg7cKO$^{DAT\text{-}Cre}$* and h*SNCA*. No gene-specific effects or interactions. n=10-15 per genotype. **(E)** Gait cadence. Two-way ANOVA for *Atg7cKO$^{DAT\text{-}Cre}$* and h*SNCA:* no gene-specific effects or interactions. n=10-15 per genotype. **(F)** Locomotor activity. Two-way ANOVA for *Atg7cKO$^{DAT\text{-}Cre}$* and h*SNCA:* main effect of *Atg7cKO$^{DAT\text{-}Cre}$*, $F_{(1,60)}$=6.64, #p=0.012. n=12-20. All data are expressed as mean ± SEM.

Hunn *et al.*, Figure S6

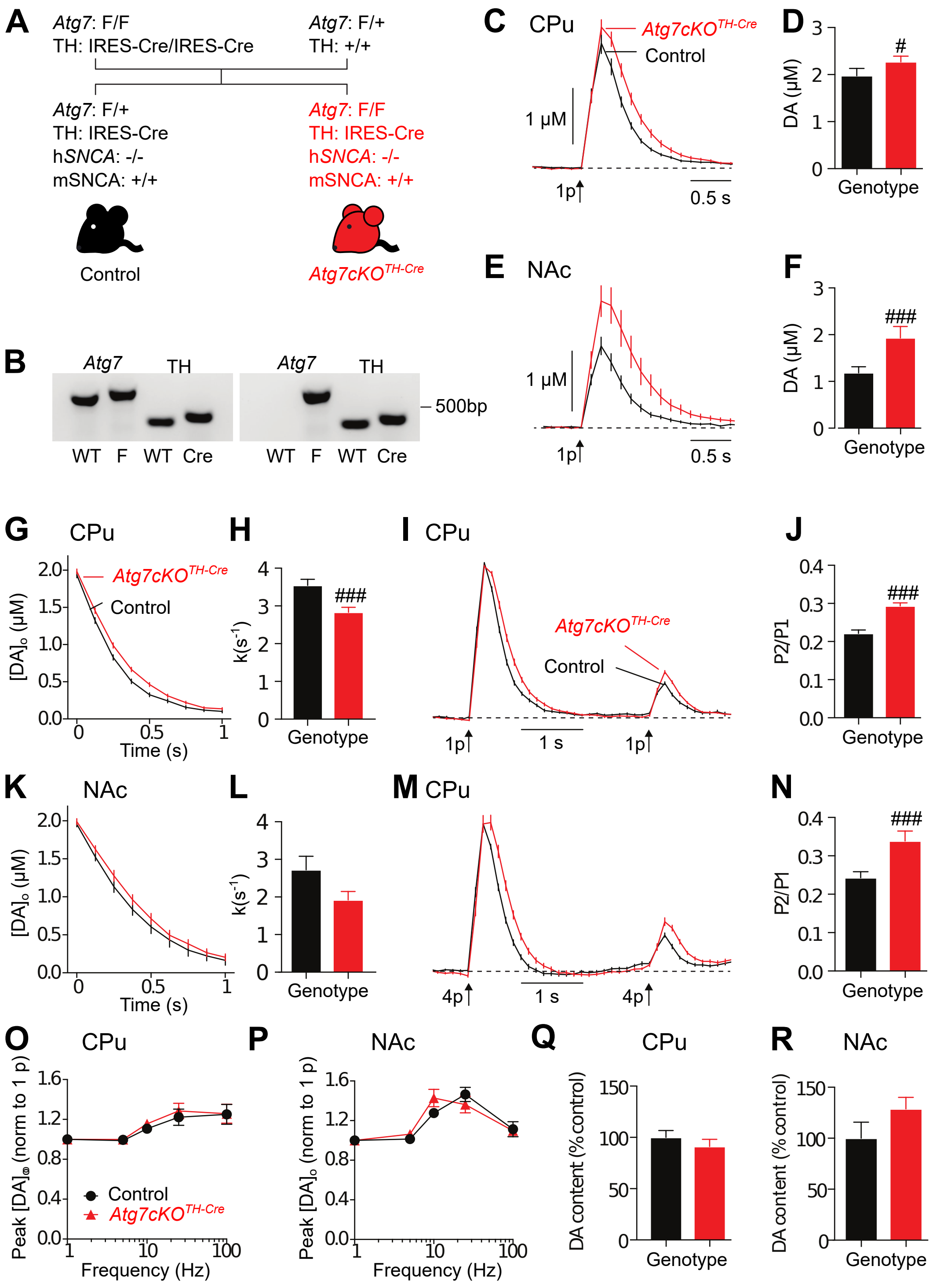

Figure S6. DA release is dysregulated when macroautophagy is impaired in dopamine neurons using a *TH*-cre driver line. Related to Figure 5.

(A) Example breeding strategy. All mice generated were heterozygous for *TH*-cre and homozygous for wild-type mouse *SNCA* (m*SNCA*). Mice had either normal (*Atg7* F/+) or impaired (F/F) macroautophagic function in dopamine neurons, and none expressed human *SNCA* (h*SNCA* -/-). **(B)** Typical PCR genotyping result using two different primer sets. *Atg7* was present as both wild-type (WT) and flanked by loxP sites ("floxed") (F). The *TH* gene was present as wild-type (WT), or driving the expression of the cre-recombinase (Cre). **(C,E)** Mean $[DA]_o$ profiles in response to a single stimulus in CPu **(C)** and NAc **(E)** in slices from 1 month-old *Atg7*cKO$^{TH\text{-}cre}$ mice. **(D,F)** Peak evoked $[DA]_o$ in CPu **(D)** and **(F)** NAc. CPu, paired *t*-test, $t(39)=2.16$, #p=0.037, n=60, 5 mice per genotype. NAc, data were log-transformed for statistical analysis, but presented as raw data for ease of comparison, paired t-test, $t(19)=4.62$, ###p<0.001. n=20, 5 mice per genotype. **(G,K)** Decay phases of evoked DA transients, concentration-matched for 2 μM $[DA]_o$ in CPu **(G)** and NAc **(K)** following maximum $[DA]_o$ at time=0 s. **(H,L)** Analysis of exponential rate constants for uptake *k,* in CPu **(H)** and NAc **(L)**. CPu only, effect of genotype, ###p<0.001. n=20-30 from 5 mice per genotype. **(I,M)** $[DA]_o$ versus time and **(J,N)** paired stimulus release ratio (P2/P1) in CPu for paired pulses **(I,J)** and paired trains (4 pulses, 100 Hz) **(M,N)**. $[DA]_o$ were enhanced in *Atg7*cKO$^{TH\text{-}cre}$ for paired pulses (*t*-test, $t(39)=5.92$, ###p<0.001) and paired trains (paired t-test, $t(39)=3.82$, ###p<0.001). n=60, 5 mice per genotype. Data were normalised to peak $[DA]_o$ at the first stimulus. **(O,P)** Peak $[DA]_o$ vs frequency for 4 pulse stimuli, normalised to 1 pulse release in CPu **(O)** and NAc **(P)**. Two-way ANOVA: no effect of genotype or interaction. n=10, 5 mice. **(Q,R)** Striatal dopamine content assessed by HPLC in CPu **(Q)** or NAc **(R)**. No effect of genotype, paired *t*-test. All data are mean ± SEM.
